# Protecting TiS_3_ Photoanodes for Water Splitting in Alkaline Media by TiO_2_ Coatings

**DOI:** 10.1021/acsami.4c07404

**Published:** 2024-06-19

**Authors:** Nuria Jiménez-Arévalo, Eduardo Flores, Alessio Giampietri, Marco Sbroscia, Maria Grazia Betti, Carlo Mariani, F. Javier García-García, José R. Ares, Fabrice Leardini, Isabel J. Ferrer

**Affiliations:** † Departamento de Física de Materiales, Universidad Autónoma de Madrid, Campus de Cantoblanco, E-28049 Madrid, Spain; ‡ Departamento de Física Aplicada, Centro de Investigación y Estudios Avanzados, 97310 Mérida, México; § Dipartimento di Fisica, Università di Roma “La Sapienza”, I-00185 Rome, Italy; ∥ ICTS-Centro Nacional de Microscopía Electrónica, Universidad Complutense de Madrid, E-28040 Madrid, Spain; ⊥ Instituto Nicolás Cabrera (INC), Universidad Autónoma de Madrid, Campus de Cantoblanco, E-28049 Madrid, Spain

**Keywords:** heterostructures, KOH corrosion, water-splitting, photoelectrolysis, hydrogen production, TiO_2_ coatings

## Abstract

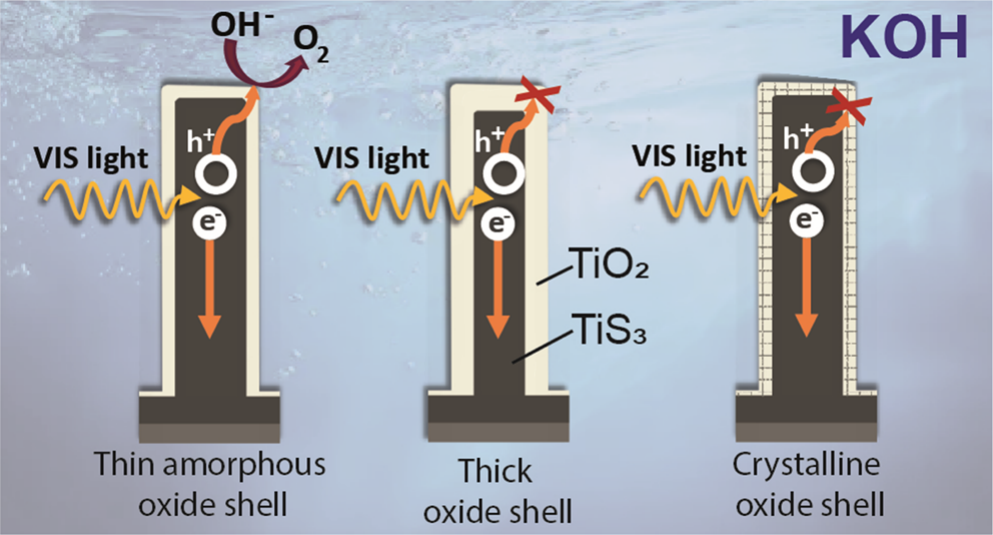

Titanium trisulfide (TiS_3_) nanoribbons, when coated with titanium dioxide (TiO_2_), can be used for water splitting in the KOH electrolyte. TiO_2_ shells can be prepared through thermal annealing to regulate the response of TiS_3_/TiO_2_ heterostructures by controlling the oxidation time and growth atmosphere. The thickness and structure of the TiO_2_ layers significantly influence the photoelectrocatalytic properties of the TiS_3_/TiO_2_ photoanodes, with amorphous layers showing better performance than crystalline ones. The oxide layers should be thin enough to transfer photogenerated charge through the electrode–electrolyte interface while protecting TiS_3_ from KOH corrosion. Finally, the performance of TiS_3_/TiO_2_ heterostructures has been improved by coating them with various electrocatalysts, NiS_*x*_ being the most effective. This research presents new opportunities to create efficient semiconductor heterostructures to be used as photoanodes in corrosive alkaline aqueous solutions.

## 1 Introduction

The increase in energy demand and the necessity to decrease polluting sources have prompted the interest in renewable energies, which face the problem of being intermittent.^1,2^ At present, new ways of storing energy from renewable sources, such as the energy storage in the molecular bonds of the hydrogen molecule, are being investigated.^3^

Photoelectrochemical (PEC) water splitting is a promising method for clean H_2_ production.^4−6^ This effect is based on the discovery of Fujishima and Honda in 1972,^7^ who reported a way to split the water molecules into oxygen and hydrogen by carrying out the photoassisted water-splitting reaction. Nowadays, much global research is focused on developing and improving the properties of semiconductor materials and electrocatalysts to perform this reaction. Some materials show great potential to be used as photoelectrodes for water splitting due to their adequate energy band gap in the VIS region and good electronic conductivity. However, some of these promising materials are unstable in aqueous media or are prone to corrosion, which limits their applicability. That is the case of titanium trisulfide (TiS_3_).

TiS_3_ is a semiconducting material of the transition metal trichalcogenide family, which grows in nanoribbons morphology with n-type conductivity. Its optical band gap has been determined to be near the VIS region (0.9–1.0 eV) by transmittance and reflectance,^8,9^ as well as by investigating its photocurrent spectral response when measured in a photoelectrochemical cell (PEC).^9,10^ Both properties, conductivity and adequate band gap, set TiS_3_ as a promising photoanode for hydrogen production. Notably, the good photoelectrocatalytic properties of TiS_3_ have been demonstrated using Na_2_SO_3_ as the electrolyte. In that media, the high photocurrents at different bias potentials, the amounts of hydrogen evolved, and the flat band potential have outlined the potential of TiS_3_ as a photoanode.^10,11^ Comparative investigations with other members of the transition metal trichalcogenide family, such as HfS_3_ or ZrS_3_, reveal that TiS_3_ is the one that outperforms in terms of photoelectrocatalytic performance.^12^ This photoelectrocatalytic activity has only been improved by substituting Nb with Ti in appropriate proportions^13^ and creating sulfur vacancies.^14^ In ref (13), an increase in the efficiency of the photogenerated hydrogen of 80% was obtained with Nb/Ti atomic ratios lower than 10%. At the same time, in ref (14), it is reported that an optimal density of sulfur vacancies of 22% improves the photocurrent by more than 2-fold. The effect of the sulfur vacancies is attributed to enhanced electron conductivity, charge separation, and transport through the charge space zone. A theoretical research^15^ by ab initio simulations shows that removing a sulfur atom increases the energy gap slightly and decreases the electron–hole coupling. The slight increase of the band gap has little influence on the electron–hole recombination rate, but the decrease of the coupling slows it significantly. These conclusions agree well with the experimental data previously reported.^14^ It is worth mentioning that these studies have been carried out in electrolytes containing Na_2_SO_3_ as a hole scavenger, which is quickly oxidated, avoiding the hole accumulation in the TiS_3_ surface and then the possibility of oxidizing it. Therefore, in the previous investigations, water splitting has not been performed. Na_2_SO_3_ was used as the sacrificial agent, so instead of water oxidation, which seems to be slower, the oxidation of Na_2_SO_3_ to Na_2_SO_4_ occurred. In the literature, no attempts to measure TiS_3_ for oxygen production have been found, and this could be due to its degradation as reported in the present work. This concern has been also based on the work of Gorochov et al.,^16^ who reported the degradation of TiS_3_ when measured in acidic and alkaline electrolytes, including KOH (the commonly used alkaline media for water oxidation). Furthermore, it should be mentioned that the corrosion of the electrode surface is even more pronounced when it acts as an anode (i.e., it is used for the oxygen evolution reaction) as TiS_3_ oxidation by the photogenerated holes can also occur. Moreover, KOH is known for being a good solution for coal desulfurization,^17,18^ which is indicative of the strong reactivity of sulfur-containing compounds with alkaline solutions. All of these hint that somehow TiS_3_ is required to be protected to generate hydrogen using KOH.

Given these challenges, recent research proved the protection against the corrosion of KOH of some semiconductors by covering them with an amorphous TiO_2_ layer deposited by atomic layer deposition (ALD).^19−23^ However, the application of ALD becomes complex when working with structured materials, such as TiS_3_. So, inspired by the possibility to fully oxidize TiS_3_ nanoribbons to TiO_2_ by a thermal annealing treatment,^24,25^ it was decided to go one step forward and improve this thermal annealing treatment to be able to control the oxidation, and thus, create TiS_3_/TiO_2_ heterostructures. To do that, an experimental setup to oxidize TiS_3_ nanoribbons was designed aiming at controlling the annealing temperature and time more precisely, while also using different oxidation atmospheres (which produced surface TiO_2_ layers with different characteristics). Once the heterostructures TiS_3_/TiO_2_ were obtained, they were characterized by a plethora of techniques, to determine their structural, morphological, chemical, and optical properties. By controlling the oxidation time and atmosphere, both the thickness and structure of the TiO_2_ layers can be tuned. Nevertheless, this TiO_2_ layer is not an effective electrocatalyst for the water-splitting reaction so, typically, electrocatalysts are deposited on top of TiO_2_ to improve the rate of the charge transfer at the electrode–electrolyte interface.^19,26^

The TiO_2_/TiS_3_ structure has been previously proposed for the visible-light photocatalytic process of contaminant degradation.^27^ In that report, the structure was created by the sulfidation of the TiO_2_/polymer nanofibers. The effect of this treatment on them was investigated, concluding that besides the TiO_2_/TiS_3_ core–shell structure, sulfur states and oxygen vacancies were formed in TiO_2_. The resulting TiS_3_-decorated TiO_2_ nanofibers showed a narrowed energy band gap of 2.3 eV (vs that of TiO_2_, 3.1 eV) and an improved efficiency for the researched photocatalytic degradation process under visible light. More recently, titanium sulfides have been proposed as attractive candidates for the photocatalytic degradation of toxic industrial pollutants such as methyl-orange and methyl-blue dyes from water waste.^28,29^ Particularly, TiS and TiS_3_ show high photocatalytic and thermocatalytic activity, exceeding that of TiO_2_ (anatase) and covering a wider range of the solar spectrum. Pristine TiS_3_ has also been successfully used to remove the mentioned pollutants under infrared irradiation. Additionally, in ref (28), the spontaneous formation of an oxide layer on the TiS_3_ active surface leading to a titanium oxide/titanium sulfide heterostructure has been observed. This layer, formed by successive photocatalytic cycles, highly enhanced the photocatalytic activity of pristine TiS_3_.

In this work, we show how the oxidation time and atmosphere can affect the structural, morphological, and optical properties of TiS_3_/TiO_2_ heterostructures. Furthermore, we tested various TiS_3_/TiO_2_ samples grown under different conditions as electrodes in KOH, comparing them to those of pristine TiS_3_. Our findings demonstrate that TiO_2_ works effectively as a protective layer against corrosion in alkaline media. Finally, different electrocatalysts were deposited on the surface of the heterostructures to enhance their performance. Overall, the results here described provide new possibilities for the rational design of adequate heterostructures for photoelectrocatalytic water splitting.

## 2 Materials and Methods

### 2.1 Synthesis

#### 2.1.1 TiS_3_ Growth

TiS_3_ samples have been obtained by the sulfurization of Ti powder (GoodFellow, 99.5%), Ti disks (15 mm diameter, GoodFellow 99.5%), and Ti thin films evaporated onto silica substrates (400 nm thick). The Ti samples and sulfur powder were sealed inside Pyrex ampules and heated at 550 °C for 20h (Ti disks and Ti thin films) and 15 days (Ti powder).^8,11^ A temperature gradient of 50 °C was created in the ampules to condense the sulfur in excess at its cold side during the cooling stage and thus avoid the contamination of the TiS_3_ samples with elemental sulfur.

#### 2.1.2 TiS_3_ Oxidation

After the TiS_3_ synthesis, the subsequent oxidation was done by introducing the specimens in a quartz tube (diameter of 20 mm), which was placed inside a tubular furnace and mounted onto two rails that allowed its movement relative to the quartz tube. TiS_3_ samples were positioned inside the quartz tube near a thermocouple to record their temperature. First, the furnace was heated to 300 °C by using a ramp of 10 °C/min. Once the temperature was stable at the target value, the furnace was moved placing the sample right at the center of the furnace. The oxidation was let run to the desired oxidation time and right after, the furnace was moved out to allow the sample to reach room temperature. Further details about the experimental procedure are given in the Supporting Information, and a detailed scheme is shown in Figure S1.

Two different atmospheres were used: air, by keeping the extremes of the tube open, and Ar flow, by connecting a gas line to the quartz tube. In the latter, a flow of 200 sccm flowed before the thermal annealing for 1 h to purge, which was reduced to 100 sccm during the oxidation procedure.

For the sake of clarity, oxidized samples are labeled in the text according to their oxidation atmosphere (air or Ar) and the oxidation time (indicated in minutes with a number). An additional table with the specific growth conditions of each sample under research in this work is given in Table S1.

#### 2.1.3 Electrocatalyst Deposition

Some electrocatalysts have been deposited/grown on the TiS_3_/TiO_2_ heterostructures that show the best performance in terms of their photocurrents (oxidation in air at 300 °C for 12 min). The electrocatalysts tested were the following: (i) Ni nanoparticles (nickel nanopowder, 99.5%, 500 nm), deposited by drop-coating them with a heptane solution; (ii) thermally evaporated Ni thin films (4 nm thick);^30^ (iii) evaporated Ni films (4 nm) sulfurized at 200 °C for 4 h;^31^ (iv) borocarbonitride (BCN) thin films deposited by the method described in refs (24, 32, and 33).

### 2.2 Characterization

The thermal stability of TiS_3_ was investigated using thermogravimetric analysis (TGA) in an SDT Q600 apparatus from TA Instruments. This instrument is coupled to a mass spectrometer Thermostar GSD 301 T3 to analyze the composition of the evolved gaseous species. The TiS_3_ powder was heated in alumina pans to a prefixed temperature ranging from 25 to 450 °C, and the mass loss was monitored. In a typical TGA run, the temperature was increased at a constant heating rate of 10 °C/min until the programmed temperature value and then the sample was cooled in ambient conditions. The experiments were performed under a flow of 90 sccm of dry air.

X-ray diffraction (XRD) patterns were recorded by using a Panalytical X’Pert Pro X-ray diffractometer at a glancing angle of incidence of 1.7°. Cu Kα radiation was used (λ = 1.5418 Å).

Raman spectra were recorded with a WiTec ALPHA 300AR, using a confocal microscope with different lenses (20× and 100×). The laser excitation wavelength was 532.3 nm with a power of 0.2 mW.

The morphology of the samples was studied by scanning electron microscopy (SEM) in a Hitachi S3000 electron microscope, coupled with an energy-dispersive X-ray spectroscopy (EDX) analyzer BRUKER Quantax (15 kV), for semiquantitative composition analysis.

Additional information about the surface composition of the samples was acquired by X-ray photoelectron spectroscopy (XPS) measurements. XPS was carried out in an ultrahigh-vacuum (UHV) chamber, with a base pressure in the low 10^–10^ mbar range. Photoelectrons, excited by a MgKα photon source (*hν* = 1253.7 eV), were measured by a hemispherical electron analyzer (VG Microtech Clam-2) in constant pass energy mode set at 50 eV for Ti and S. Calibration of the binding energy (BE) position with respect to the Fermi level for the observed lines was done by acquiring the Au 4f_7/2_ (84.0 eV of BE) core level after each measurement. The data acquisition was done after long annealing of the samples at 110 °C from 4 h up to overnight (depending on the initial cleanliness of the sample surface). The low annealing temperature was chosen to avoid altering the surface composition and to get rid of water and other atmospheric pollutants, as it will be justified by the TGA results.

High-resolution transmission electron microscopy (HR-TEM) studies were carried out in a JEOL 2100HT operated at 200 kV, using magnifications of 40–50 k. The scope is attached to a Gatan Orius SC1000 CCD camera for recording the images digitally and an OXFORD INCA detector for X-ray energy-dispersive spectrometry (XEDS) compositional analysis. High-resolution images, as well as selected area electron diffraction (SAED) patterns, have been recorded. As-obtained samples were scrapped with a scalpel to remove some nanoribbons away from the Ti disk used as substrate. These were broken and dispersed in butanol using an ultrasonic bath. One drop of the resulting dispersion was put onto amorphous carbon holey layer-coated Cu grids with a pipet.

The optical density (OD) of the samples was investigated using samples grown on silica substrates. The transmittance spectra were recorded with a double-beam ultraviolet–visible (UV/vis)/NIR Lambda 1050 PerkinElmer spectrometer (190–1800 nm of wavelength range). This equipment has a spot size of 5 mm^2^. The optical density of the silica substrate was also obtained by measuring its transmittance spectrum and was then subtracted from the optical density of the samples grown onto these substrates.

Diffuse optical reflectance spectra were recorded in a UV/vis/NIR PerkinElmer LAMBDA 950 spectrophotometer equipped with an integrating sphere to collect the reflecting radiation in the 300–2000 nm spectral range. A spot size of 21 mm^2^ was used.

Photoelectrochemical measurements were made in a three-electrode cell. The TiS_3_ and TiS_3_/TiO_2_ samples, with an apparent area of 1.3 cm^2^, were used as the working electrode (WE), a platinum foil of 9 cm^2^ was used as the counter electrode (CE), and an Ag/AgCl electrode filled with 1.0 M KNO_3_ solution (XR440 from Radiometer Analytical) was used as a reference electrode (RE). This RE has an electrode potential *E*_Ag/AgCl_^0^= 484 mV vs normal hydrogen electrode scale (*E*_NHE_). These three electrodes were immersed in 0.1 M KOH aqueous solution (pH 13.0), and an Ar flow (20 sccm) was bubbled through this electrolyte during the experiments. More details about the photoelectrochemical experimental system can be found in the Supporting Information (Figure S2).

Measured electrode potentials (*E*_Ag/AgCl_) have been converted to the reversible hydrogen electrode scale (*E*_RHE_) using eqs 1 and 2.
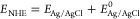
1
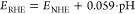
2

To investigate the photoresponse of the electrodes, chronoamperometry measurements at a fixed potential were made in the dark and under illumination by using a potentiostat-galvanostat PGSTAT302N (Autolab) provided with an integrated impedance FRA II module (Figure S2). The WE was illuminated with a halogen lamp (Osram 650 W). The intensity of light reaching the sample was 65 W/m^2^.

In addition, electrochemical impedance spectroscopy (EIS) measurements have been done to characterize the electrolyte-semiconductor interface by using a sinusoidal AC voltage signal with an amplitude of 10 mV and a variable frequency between 100 and 1000 Hz superimposed to the bias potential.

## 3 Results and Discussion

### 3.1 Thermal Oxidation Analysis

We first investigated the thermal oxidation of TiS_3_, aiming at controlling the growth of the TiO_2_ shells at the surface of TiS_3_ nanoribbons. For this purpose, TGA measurements were conducted by heating the TiS_3_ powder in a dry air atmosphere up to different temperatures. Simultaneously, the gases evolved during the thermal treatment were analyzed by concomitant mass spectrometry (MS) measurements.

**Figure 1:**
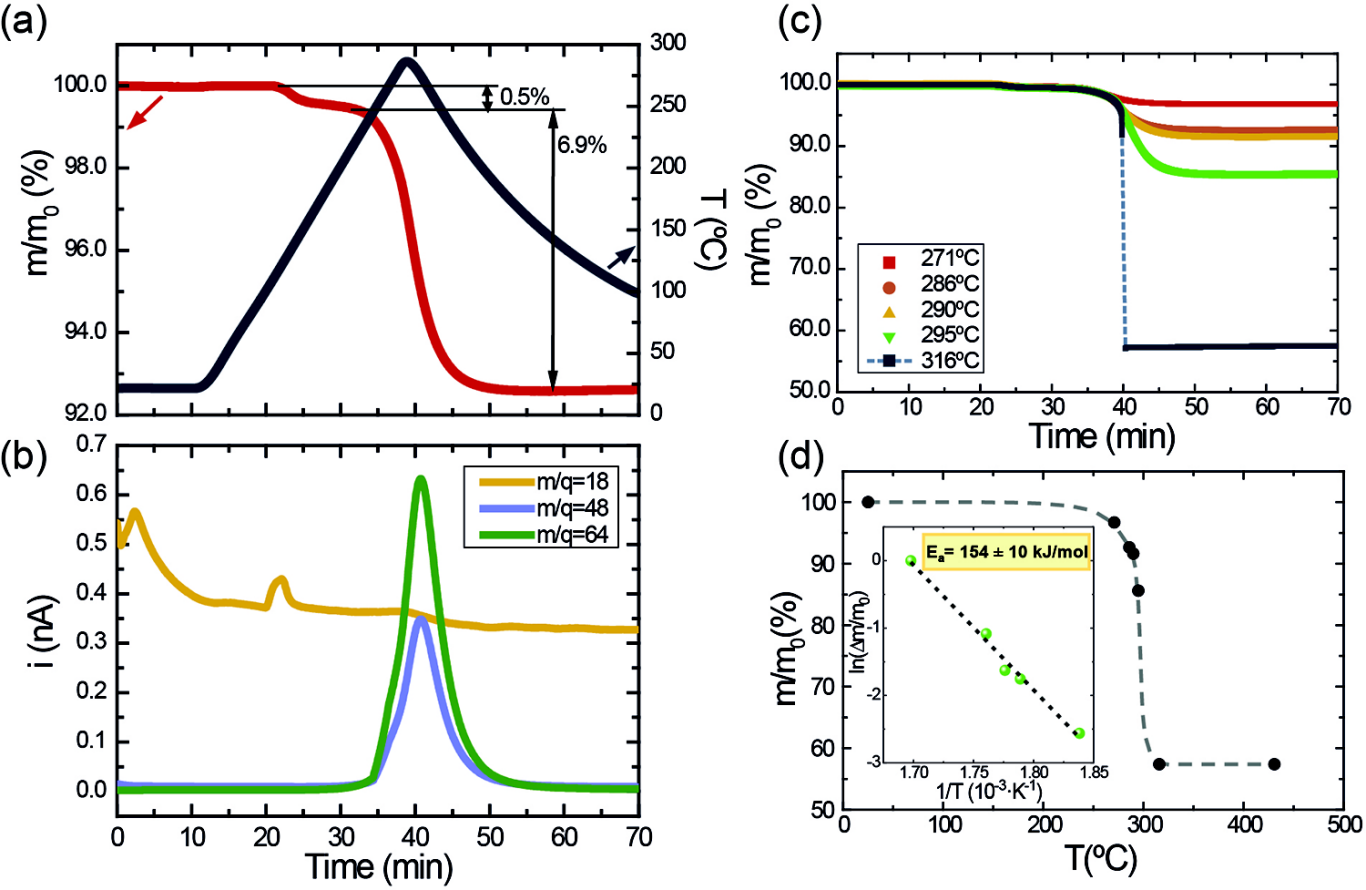
(a) TGA curve of TiS_3_ showing the time evolution of the relative mass (left axis) and temperature (right axis). (b) Mass spectrometric ionic currents (recorded at *m*/*q* = 18, 48, and 64) as a function of time, measured during the TGA experiment. (c) Relative mass vs time at different applied temperatures. (d) Relative mass of TiS_3_ samples when heated at different temperatures between ambient temperature (25 °C) and 450 °C; inset: representation of the Arrhenius equation.

The typical mass loss curve of TiS_3_ recorded by TGA (Figure 1a,b) shows the mass ionic currents observed at *m*/*q* = 18, 48, and 64, as a function of time, recorded during the TGA run. Those signals indicate that there is a first mass loss event at temperatures below 135 °C that corresponds to H_2_O desorption (*m*/*q* = 18), justifying that heating at 110 °C for the XPS measurements is enough to get rid of water and other pollutants without altering the composition of the sample. Next, there is a second mass loss around 270 °C that is accompanied by the release of SO_2_ molecules (seen at *m*/*q* = 48 and 64). These results indicated that TiS_3_ oxidation occurs following this reaction
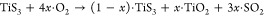
3

Based on these TGA-MS measurements done using different heating temperatures, we determined that TiS_3_ oxidation starts at around 270 °C (Figure 1c), placing the range that might be used for the oxidation treatment between 270 and 315 °C. The fraction of TiS_3_ converted into TiO_2_ can be obtained from the residual mass obtained after heating the samples (see Figure 1d). TGA results indicate that the sample oxidized at the higher temperature presents a residual mass of 57.4%, which is close to the expected residual mass obtained by the total conversion of TiS_3_ to TiO_2_ (56%).

The activation energy (*E*_a_) of this reaction can be obtained by using Arrhenius equation (eq 4), in which *k* is the rate constant, *A* is a pre-exponential factor, *R* is the ideal gas constant, and *T* is the temperature.^34^
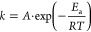
4

So, an Arrhenius plot of the converted fraction as a function of the inverse of the maximum applied temperature allows the activation energy of the TiS_3_ oxidation reaction to be obtained (Figure 1d, inset). The resultant *E*_a_ has a value of 154 ± 10 kJ/mol, which is comparable to and in good agreement with the ones obtained in the oxidation of TiS_3_ under an Ar flow,^35^ TiS_2_ and Ti_2_S_3_,^36^ and other sulfides.^37,38^

### 3.2 Structural, Morphological, and Compositional Characterization of the TiS_3_/TiO_2_ Heterostructures

**Figure 2:**
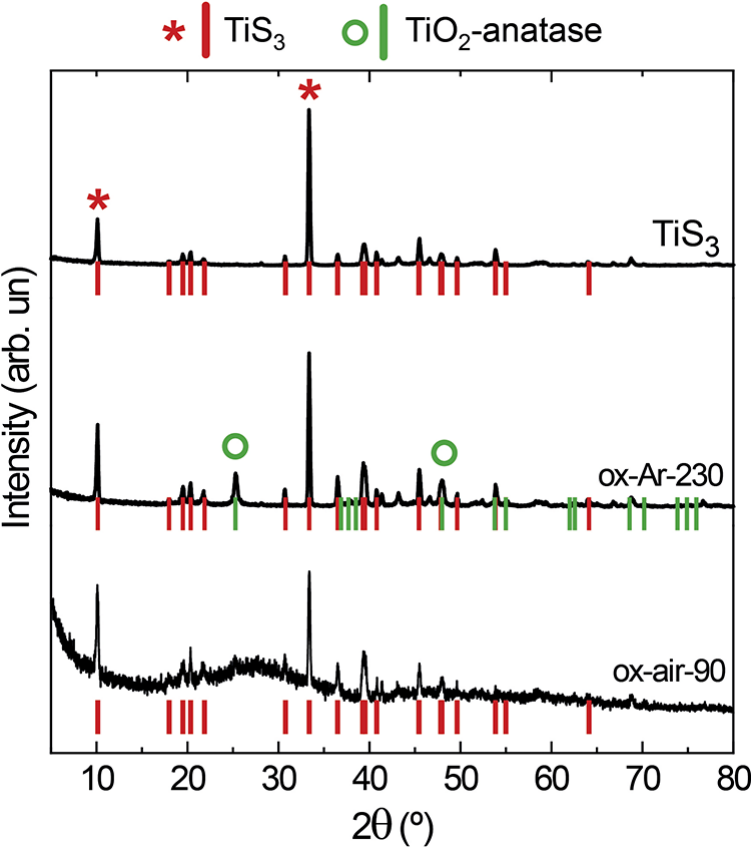
XRD diffractograms of three samples: TiS_3_, ox-Ar-230, and ox-air-90. TiS_3_ and TiO_2_-anatase peaks correspond to PDF 00–015–0783 and PDF 01–071–1167 files, respectively. The most intense peaks of TiS_3_ and TiO_2_-anatase are indicated with a red asterisk and a green circle, respectively.

The effect of annealing treatment and the atmosphere used to oxidize TiS_3_ was investigated by XRD (Figure 2). Samples treated under an Ar atmosphere (ox-Ar-230) showed the coexistence of monoclinic TiS_3_ and tetragonal TiO_2_. The latter has been assigned to the anatase structure type, whose more intense peaks are at 2θ of 25 and 48°. On the other hand, the sample oxidized in air (ox-air-90) shows, in addition to the TiS_3_ peaks, a wide peak centered at 2θ = 28°, which might suggest the formation of amorphous TiO_2_.^39−41^ Further evidence of the presence of amorphous TiO_2_ structure is given below, based on Raman and SAED analyses.

**Figure 3:**
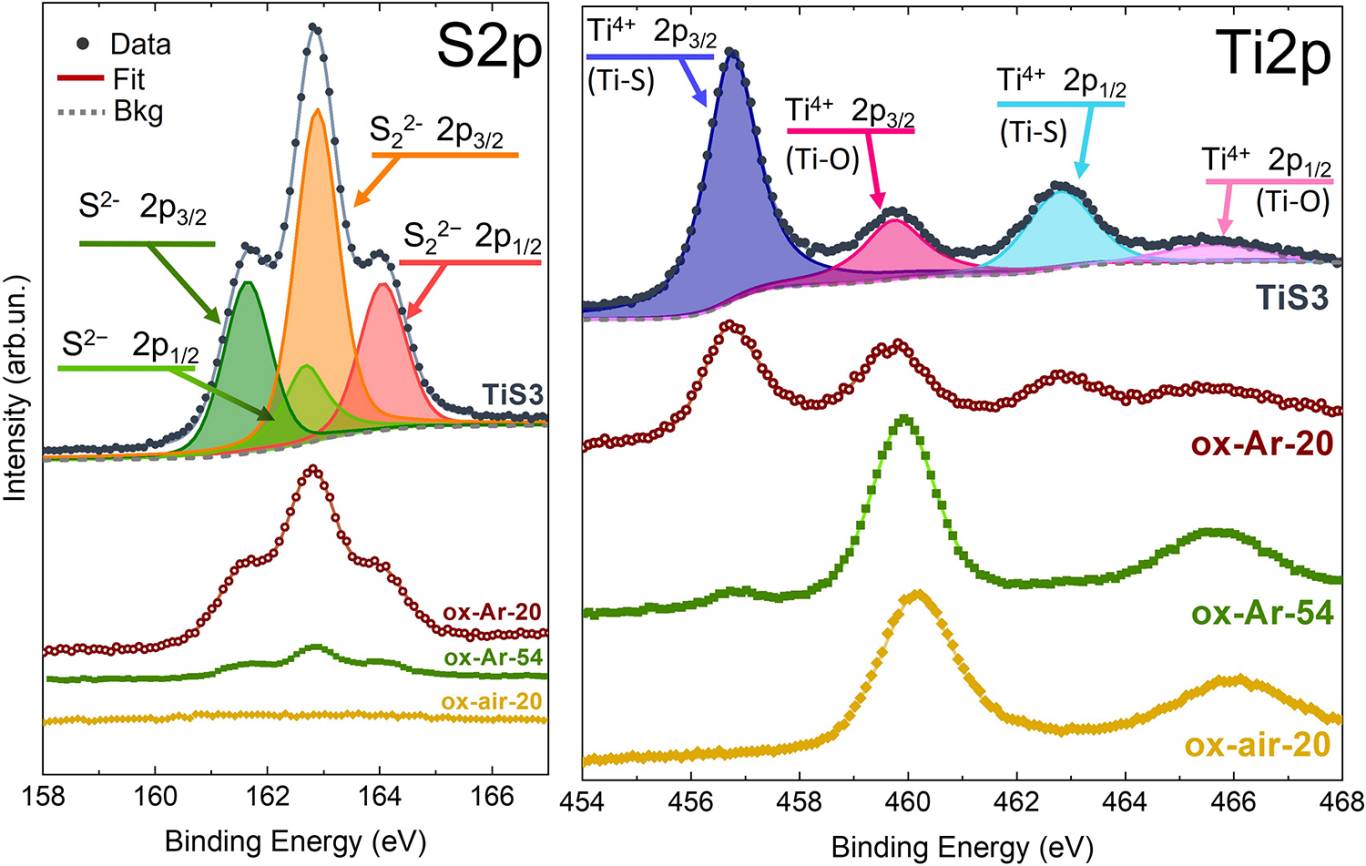
S 2p (left) and Ti 2p (right) XPS spectra for TiS_3_, ox-Ar-20, ox-Ar-54, and ox-air-20 samples; the data are stacked along the vertical axis for clarity. Experimental data (dotted lines), single fitting curves (colored continuous lines), and total fitting (continuous black line). The single fitting curves and total fitting are shown only for the TiS_3_ case.

Additional characterization of the chemical bonding state at the surface of the samples was carried out by XPS. Figure 3 shows the S 2p and Ti 2p core-level spectra for pristine TiS_3_ and three oxidized samples, 20 min in argon (ox-Ar-20), 54 min in argon (ox-Ar-54), and 20 min in air (ox-air-20). The curves were analyzed and fitted using pseudo-Voigt line shapes (Lorentzian–Gaussian curves associated with the intrinsic excitation lifetime and the overall experimental uncertainty, respectively) after subtracting a Shirley background as a fitting parameter. For the sake of simplicity, we report only the complete fitting of the TiS_3_ spectrum in Figure 3, while the fittings of all of the measurements of the treated samples and the fitting parameters obtained can be found in Figure S3 and reported in Table S2, respectively.

The S 2p core level for TiS_3_ is composed of two spin–orbit split (S 2p_3/2_ and S 2p_1/2_) doublets, corresponding to S^2–^ and S_2_^2–^ sulfur species.^42−44^ The intensity of the peaks decreases with the oxidation time, as we can see by the gradual intensity reduction from the pristine sample to the ox-Ar-20 until the ox-Ar-54. The ox-air-20 sample does not present any peak ascribed to the S^2–^ or S_2_^2–^, which suggests the absence of any sulfur bond assigned to the TiS_3_ on the topmost layers of the nanoribbons after oxidation in air.

The Ti 2p core level for TiS_3_ also manifests two spin–orbit split doublets, at 456.8 and 462.8 eV (Ti 2p_3/2_ and Ti 2p_1/2_, respectively), corresponding to the Ti^4+^ component characteristic of Ti–S bonds;^42−44^ the other Ti^4+^ doublet at 459.7 and 465.6 eV (Ti 2p_3/2_ and Ti 2p_1/2_, respectively) can be assigned to the formation TiO_2_.^45^ This suggests that the surface of our TiS_3_ samples might be slightly oxidized, even in the pristine state, before any intentional oxidation, due to residual ambient contamination. However, we demonstrate that the Ti^4+^ peaks ascribed to the Ti–S bonds diminish after thermal oxidation, whereas the ones owing to the Ti–O bond increase. In particular, the ox-air-20 sample is composed of just TiO_2_ on its surface. Nevertheless, it is worth mentioning that XPS is an experimental technique sensitive to the topmost few layers of the sample. Therefore, despite not seeing Ti–S bonds by this technique, the presence of TiS_3_ in a sample oxidized for 90 min in air was still observed by XRD (see Figure 2).

Thus, the XPS core-level measurements prove that we can control the thickness of the oxide layer with oxidation time. In addition, it is noticed that the oxidation in air occurs faster than that in Ar. Further evidence of this finding is given below by the results obtained by optical transmittance measurements (see Section 3.3).

Moreover, an additional peak around 170 eV is appreciated in the ox-air-20 sample (see Figure S4), due to the formation of sulfates.^46−48^

**Figure 4:**
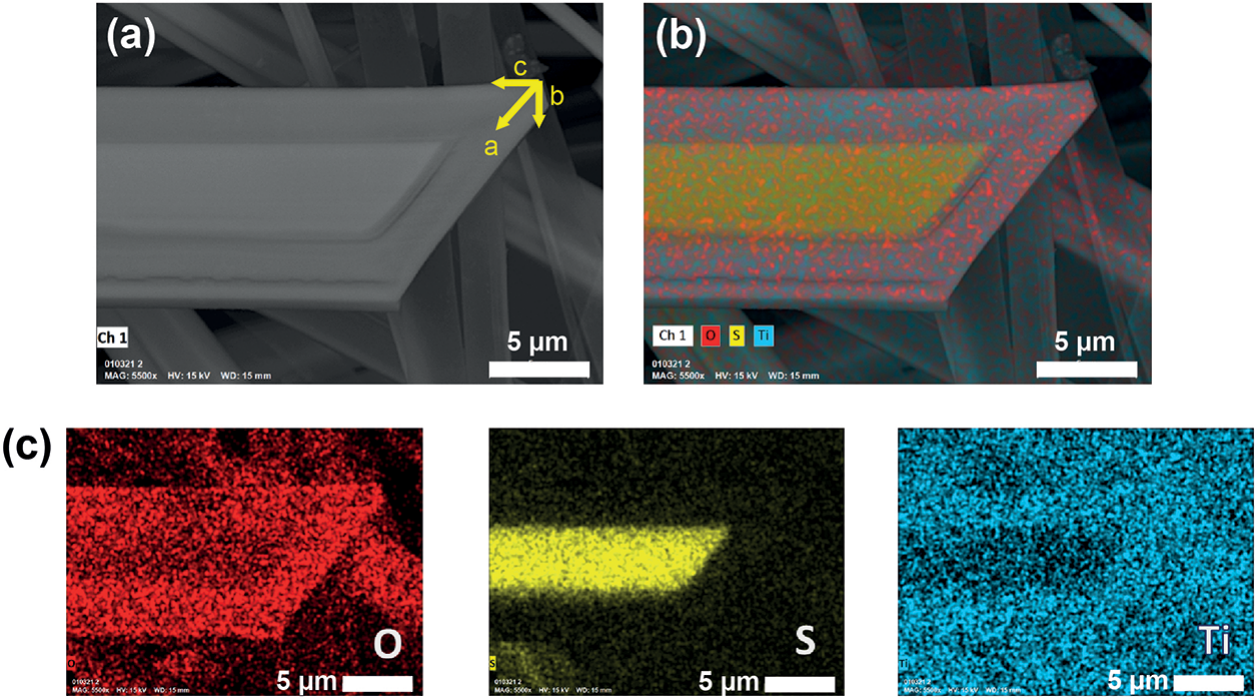
(a) SEM image of sample ox-air-90. (b) EDX mapping analysis superposed with the image in (a). (c) EDX mapping results for oxygen (red), sulfur (yellow), and titanium (blue).

The previous results describe the oxidation at the macroscopic scale. To deeply understand how oxidation occurs in the nanoribbons, we performed SEM and EDX analysis. SEM images (Figure 4a) showed a contrast between the shell and the core of the nanoribbons after the oxidation procedure. EDX mapping analysis (Figure 4b,c) revealed a higher concentration of oxygen along the edges, whereas sulfur appears only in the core of the nanoribbon. Regarding the Ti mapping, a lower signal in the center of the ribbon is evident compared to the verges, which can be ascribed to a higher density of Ti in TiO_2_ (shell) compared to TiS_3_ (core). Further information about the density of Ti in both phases can be found in the SI (Table S3).

Moreover, from the compositional EDX mapping, it can be concluded that the oxidation process is anisotropic. The nanoribbons are preferentially oxidized along the *a* and *c* directions (see Figure 4). This experimental observation suggests that oxidation occurs through layers and not between layers. This result is supported by the fact that TiS_3_ nanoribbons have a layered morphology,^49^ which favors the formation of a core–shell-like structure.

**Figure 5:**
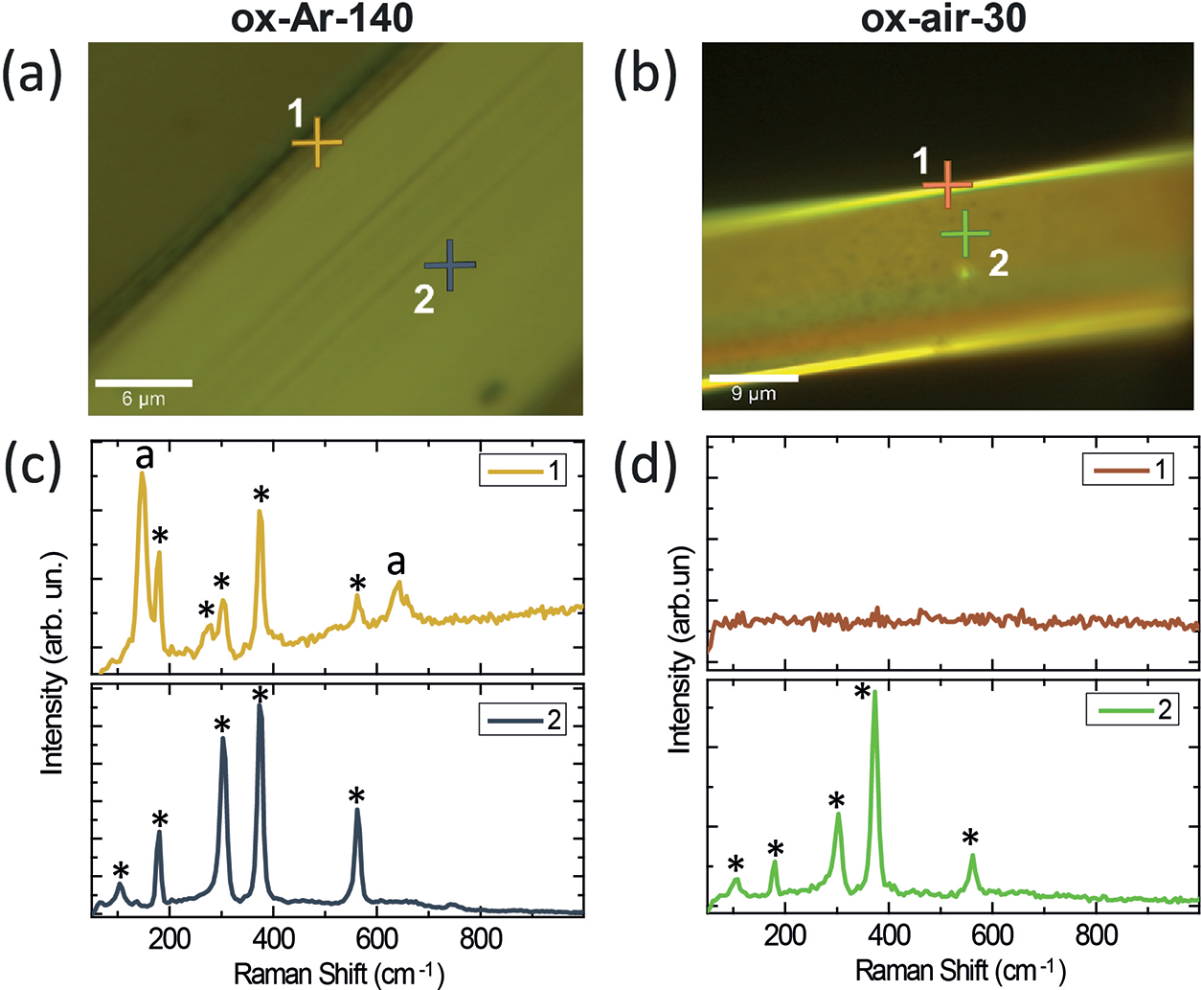
Optical microscopy images of (a) ox-Ar-140 and (b) ox-air-30 samples. (c) Raman spectra in the edge and the center of one ribbon of the ox-Ar-140 sample (marked with 1 and 2 in (a), respectively). (d) Raman spectra for the ox-air-30 sample in the edge and the center of one ribbon (marked with 1 and 2 in (b), respectively). TiS_3_ peaks are indicated with *, and anatase TiO_2_ peaks are indicated with “a”.

Raman spectra were measured at the edges and at the center of the nanoribbons in samples oxidized under argon flow (Figure 5a,c) and in samples oxidized in air (Figure 5b,d). Independently of the atmosphere used, the center of the nanoribbon (labeled 2 in Figure 5a,b) shows the typical spectrum for the TiS_3_ compound (showing peaks at 103, 175, 300, 370, 563 and 730 cm^–1^).^24,50^ In contrast, differences are clearly visible in the spectrum obtained in the oxide shell (labeled 1 in Figure 5a,b). Raman spectrum in the edge of the sample oxidized in argon (Figure 5c), suggests the presence of anatase TiO_2_ (peaks at 145 and 640 cm^–1^).^24,51,52^ This last spectrum also shows the appearance of a peak at 270 cm^–1^, which we assigned to TiS_3_. This peak is only observed at the edges of the TiS_3_ nanoribbons due to the different symmetry of the crystalline planes observed in this region.^53^ Further evidence on the nature of the 270 cm^–1^ Raman band are shown in Figure S5, where the intensity of the TiS_3_ Raman peaks along the width of a single nanoribbon are shown for the first time. On the other hand, the Raman spectrum at the edge of the air-oxidized sample only presents the zero Raman shift peak (i.e., the elastic reflection of the incident laser). This indicates that the spectrum was recorded at the sample edge and not out of the sample. Apart from that peak, the Raman spectrum does not show any other signal, suggesting that there is a layer with an amorphous structure.

**Figure 6:**
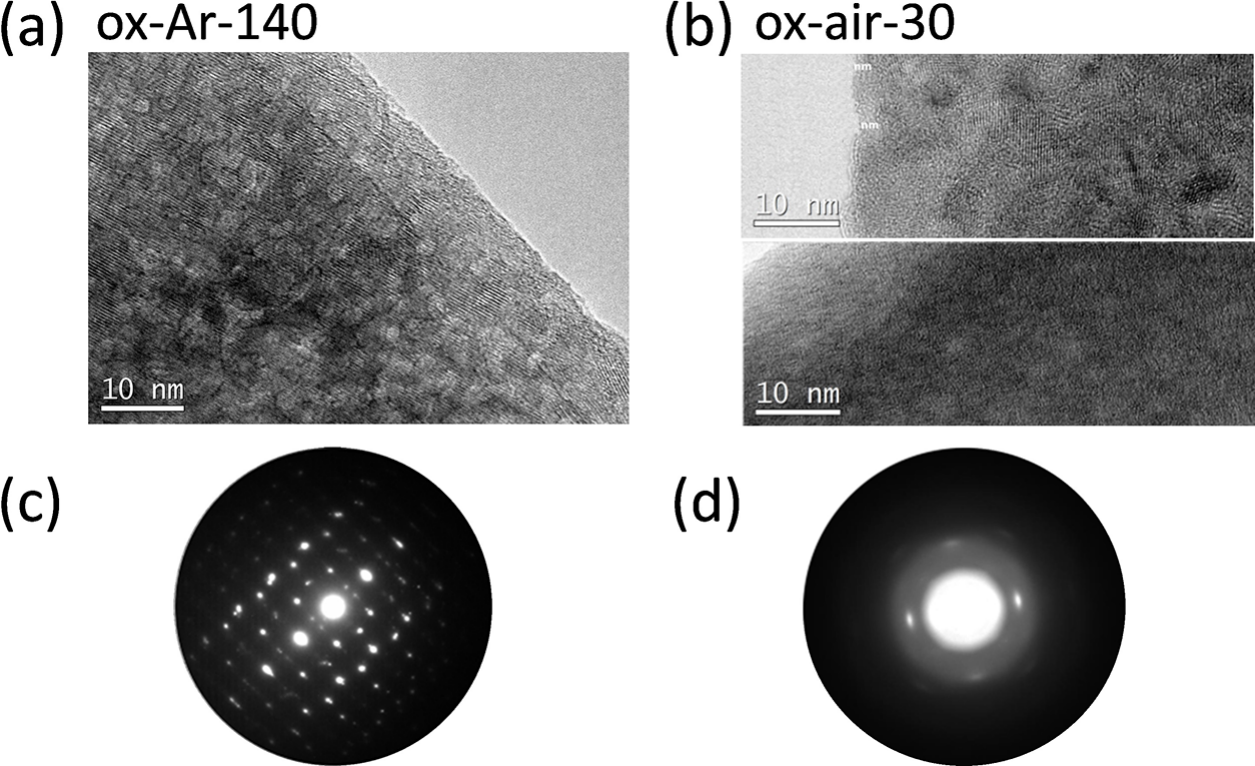
(a, b) Collection of high-resolution TEM images recorded in ox-Ar-140 and ox-air-30, respectively, and (c, d) the corresponding electron diffraction patterns. They are oriented with respect to the images and the spacing for the most prominent reflections is included.

High-resolution TEM images were also used to investigate the morphology and structure of samples oxidized in Ar and in air atmospheres (ox-Ar-140 and ox-air-30), as shown in Figure 6a,b, respectively. In Figure 6c,d, the corresponding SAED patterns, recorded in the same regions as the images, have been included. Concerning the structural properties of the samples, there is a clear difference between both samples. In Figure 6a, for the ox-Ar-140 sample, there are some nanosized crystalline domains with tiny amorphous spaces in between them. Note that the crystalline fringes are all well aligned so that some coalescence seems to happen. Besides, some porous-like spaces between the crystalline domains were observed. Figure 6b shows two different parts of sample ox-air-30. Figure 6b (top) shows details of a particle in an area where highly crystalline nanodomains exist. In clear contrast, Figure 6b (bottom) shows a different area of the same particle, where the structure is more amorphous, even though some small domains exhibit lattice fringes. The corresponding diffraction pattern of ox-air-30 (Figure 6d) gives the impression of a much more amorphous material than that of ox-Ar-140 (Figure 6c), obviously because of the presence of significant amorphous areas. It can be noted that the measured SAED patterns fully agree with the XRD and Raman results, demonstrating once again that oxidizing under an Ar flow leads to a crystalline structure, whereas oxidizing in air promotes the formation of an amorphous shell.

Altogether, the results obtained by different structural, morphological, and compositional techniques permit deducing that the oxidation of TiS_3_ samples occurs at the edges of the nanoribbons forming a core–shell structure. The TiO_2_ shell is amorphous if oxidized in air or crystalline if the thermal annealing is performed under an Ar flow with a residual quantity of O_2_. In addition, it has been revealed that the longer the thermal annealing treatment is, the thicker the oxide shell becomes. It has been demonstrated that the oxidation kinetics are not equivalent in each atmosphere, being significantly slower when oxidizing in Ar, as only a residual amount of oxygen is present during oxidation. This causes the oxidation process to stop at some point when oxidizing in Ar, as oxygen is consumed, whereas it is not limited in air.

### 3.3 Optical Characterization of the TiS_3_/TiO_2_ Heterostructures

Optical characterization of the TiS_3_/TiO_2_ heterostructures was done to get a deeper understanding of how the thickness of the oxide layer affected the optical density (OD), reflectance (*R*), and energy band gap of the samples. For that aim, two types of experiments were done.

**Figure 7:**
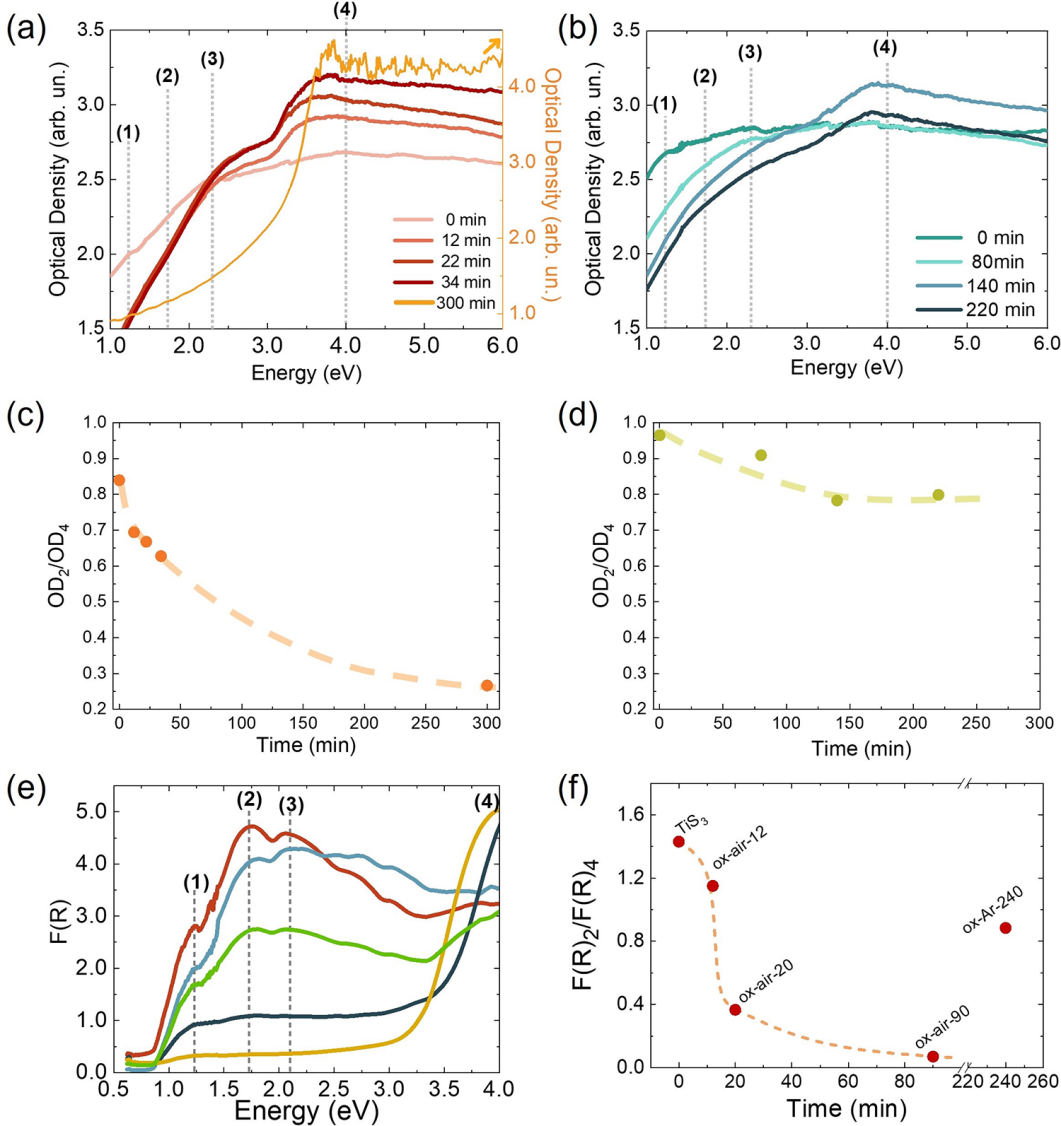
Optical density spectra obtained from transmittance measurements of TiS_3_ samples grown on fused silica and oxidized (a) in air (right axis in orange corresponds just to the OD of the sample oxidized for 300 min) and (b) under Ar flow for different times. Ratio between optical density values of the (2) and (4) bands (OD_2_/OD_4_) against the oxidation time for samples oxidized (c) in air and (d) under Ar flow whose OD curves are shown in (a, b). (e) Kubelka–Munk function obtained from diffuse reflectance measurements for samples TiS_3_ (red), ox-air-12 (light blue), ox-air-20 (dark blue), ox-air-90 (yellow), and ox-Ar-240 (green) as a function of the incident photon energy. (f) Relationship between *F*(*R*)_2_ over *F*(*R*)_4_ as a function of the oxidation time. Corresponding samples to the data are labeled in the figure. Dashed lines in c, d, and f are used as visual guides.

First, optical transmittance measurements were performed on TiS_3_ samples grown on fused silica and oxidized for different times in air and Ar atmospheres (see Figure 7a,b), in order to determine the optical density using Beer’s law.^54^ Pristine TiS_3_ has three absorption bands (1.23, 1.73, and 2.10 eV marked as (1), (2), and (3) in Figure 7a,b).^8,24^ These bands are more clearly visible by diffuse reflectance measurements (Figure 7e). It can be observed that the OD in the region of TiS_3_ absorption diminishes as the oxidation time is increased for both atmospheres, decreasing more abruptly in the case of air-oxidized samples (Figure 7a) than in Ar-oxidized samples (Figure 7b), which diminish softly. Moreover, it can be appreciated that a band (marked as (4)) starts to arise in the UV region assigned to TiO_2_.^24^

To further investigate the effect of the oxidation of TiS_3_ and its dependence on the atmosphere used, the ratio of the OD values of bands (2) and (4) has been compared, since these bands are due to TiS_3_ and TiO_2_, respectively (Figure 7c,d). It can be appreciated that, for both atmospheres, the longer the sample is under the thermal annealing treatment, the more oxidized the sample is (i.e., OD_2_/OD_4_ decreases). On the other hand, if the two atmospheres are compared, it can be concluded that the oxidation in air occurs faster than in Ar, as would be expected by taking into account that the concentration of oxygen in air is 21%, whereas, in the Ar treatment, samples are oxidized with a residual quantity of oxygen present in the argon flow. In particular, for two samples oxidized at similar times (300 min in air and 220 min in Ar), it can be noticed that the OD_2_/OD_4_ ratio is 3.5-fold smaller in air-oxidized samples, thus indicating that there is more oxide. Furthermore, saturation of the OD_2_/OD_4_ parameter in Ar can be observed (Figure 7d), probably due to the consumption of the residual oxygen and its displacement by the constant Ar flow, which causes the oxidation process to stop.

Second, diffuse reflectance measurements were done on the samples explored as electrodes for the OER in the next section in order to understand the influence of the oxide shell on their optical properties and relate these to the photoelectrochemical performances. These samples were grown over Ti substrates, which makes impossible the analysis of their OD by measuring the transmittance. Therefore, the diffuse reflectance (*R*) spectra were obtained, and the R data were converted into Kubelka–Munk function *F*(*R*), which is proportional to the OD.^55^

Figure 7e shows the *F*(*R*) as a function of the photon energy. TiS_3_, ox-air-12, and ox-Ar-240 samples exhibit the typical TiS_3_ absorption bands (marked as (1), (2), and (3) in the figure).^8,24^ These bands, and thus the absorption in the visible range, decrease as the degree of oxidation is increased, whereas the bands in the UV region (assigned to TiO_2_) start to arise. This effect is the same as that observed before with the optical density measurements in Figure 7a,b.

For these samples, a quantitative estimation of the oxidation degree was also obtained from the ratio between the value of *F*(*R*) of the TiS_3_ peak (2) and the value of *F*(*R*) at 4.0 eV (peak (4), defined as the reference for TiO_2_). This ratio is used later in the photoelectrochemical discussion. Results as a function of oxidation time are shown in Figure 7f. It is evidenced, again, that when the oxidation time is increased, the oxide layer grows and starts to absorb in the UV region, whereas the relative amount of TiS_3_ decreases. Moreover, it is appreciated that the sample oxidized in Ar (ox-Ar-240) shows higher *F*(*R*) ratio values similar to samples oxidized in air for 10–20 min, consistent with the OD analysis done before.

The *F*(*R*) plot suggests that a heterostructure with two optical band gaps has been created. Tauc fitting equation for direct band gaps ((*F*(*R*).*h*ν)^2^ vs *h*ν)^56^ has been applied to the *F*(*R*) function (see Figure S6). The low band gap in all of the samples has an average value of 0.96 ± 0.03 eV and does not change with the oxidation time or atmosphere (see Table S4). This band gap agrees with the reported value for TiS_3_.^8,24^ It is difficult to accurately determine the exact band gap value for the TiO_2_ shell from those measurements, but it is estimated to be around 3.2 eV in accordance with the value of TiO_2_ anatase nanoribbons.^8,57^ It can then be concluded that this TiS_3_/TiO_2_ heterostructure has two direct band gaps, which are more evident in the more oxidized samples (ox-air-20 and ox-air-90), as shown in Figure S6a.

### 3.4 Testing TiS_3_/TiO_2_ Heterostructures as Photoelectrodes for the OER

For decades, transition metal sulfides have been proposed as electrocatalysts for water decomposition and hydrogen evolution. However, two challenges persist: first, their low efficiency, and second, their limited stability over time. To date, the strategies proposed to face these challenges are mainly focused on modifying either the nanostructure or the electronic configuration.^58^ Additionally, strategies based on the control of the morphology have also been proposed for metal sulfide- and phosphide-based nanocatalysts.^59^

In this work, the photocatalytic response of TiS_3_ samples in their pristine state (without optimizing treatments) was investigated in alkaline electrolyte (KOH) besides the effect of protecting them from oxidation by covering them with TiO_2_ layers. Four TiS_3_ samples oxidized under different conditions (time and atmosphere) were tested as electrodes in a PEC to understand how the thickness and structure of the oxide layer affected the photoelectrochemical behavior. The selected samples are those characterized by diffuse reflectance (Figure 7e,f).

**Figure 8:**
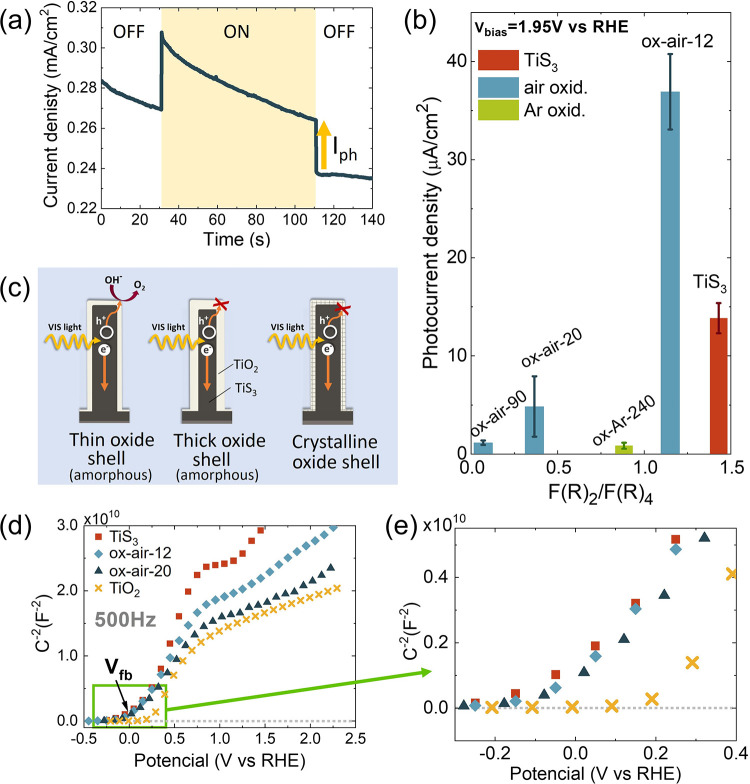
(a) Chronoamperometry measurement at 1.95 V vs RHE for ox-air-12 in the dark (OFF) and light (ON). (b) Photocurrent as a function of the oxidation degree estimated by the ratio *F*(*R*)_2_/*F*(*R*)_4_, described in the optical characterization section. (c) Schematic diagram of the formation of the electron–hole pair and the pathway of the hole to the surface of the heterostructure. (d) Mott–Schottky plots for TiS_3_, ox-air-12, and ox-air-20 at 500 Hz. TiO_2_ data has been included from ref (24). (e) Zoom to the region closer to C^2–^ = 0 F^–2^.

First, the influence of the TiO_2_ shell in the photoelectrochemical properties of the TiS_3_/TiO_2_ heterostructures was evidenced by measuring the photocurrents. Figure 8a shows a representative chronoamperometry measurement at a bias potential of 1.95 V vs RHE for the sample ox-air-12 in the dark and under illumination with a halogen lamp. It can be observed that the photocurrent (*I*_ph_) has a positive value in good agreement with the behavior of TiS_3_ as an n-type semiconductor.^11^ The existence of photocurrent under VIS illumination in an oxidized sample hints that the electron–hole pairs are photogenerated in the TiS_3_ core, as TiO_2_ is transparent to visible light. It can be noted that the thickness and structure of the oxide shell play important roles in the value of the photocurrent (Figure 8b).

Regarding the thickness of the TiO_2_ layer, a general trend is noted in which the more oxidized the sample is, the lower photocurrent exhibits. However, a very short oxidation process leads to a higher (3 times) photocurrent than pristine TiS_3_. Figure 8b shows the photocurrent against *F*(*R*)_2_/*F*(*R*)_4_, a parameter used previously to estimate the oxidation degree of the electrodes. This fact hints that the photogenerated holes in TiS_3_ must go through this oxide shell to reach the electrode/electrolyte interface and oxidize water. Therefore, although the TiO_2_ layer is optically transmissive, its presence could facilitate the recombination of the electron–hole pairs if it is not thin enough or its conductivity is low. Here, it is worth mentioning that the conductivity of TiO_2_ is clearly dependent on the oxygen vacancy density.^60^Figure 8c shows a scheme to exemplify this behavior.

On the other hand, the structure is also critical. It is observed that although ox-Ar-240 has a thinner oxide shell than other samples oxidized in air (see Figure 7f), its photocurrents are lower. This is due to the crystalline structure of this TiO_2_ layer (anatase), whose conductivity is 4-fold lower than that of the amorphous TiO_2_^61^ known as a good hole conductor.^19^ Furthermore, it is known that the high defect density (mainly oxygen vacancies) existing in an amorphous TiO_2_ layer enhances the carrier conductivity, charge separation, and transport through the charge space zone to the electrolyte interface improving the efficiency of the charge transfer.^62^ As a result, the photocatalytic properties are upgraded as mentioned above for the ox-air-12 samples versus pristine TiS_3_.

Moving away from these considerations, different approaches can be employed to obtain the flat band of a semiconductor used as an electrode in a PEC.^20^ Among them, the Mott–Schottky plot is a well-established method to determine the semiconductor flat band potential which is obtained by measuring the electrochemical impedance of the semiconductor/electrolyte interface by EIS.^64,65^ From these measurements, the capacitance of the space charge zone in a semiconductor is determined, and the inverse of its square (*C*_SC_^–2^) is plotted vs the applied potential (*V*_bias_). If this plot can be described by the Mott–Schottky equation (eq 5), then the potential at which (*C*_SC_^–2^) is zero corresponds to the flat band potential.

The Mott–Schottky equation is
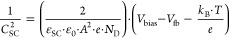
5where *C*_SC_ is the semiconductor capacitance at the interface; ε_SC_ and ε_0_ are the semiconductor and vacuum dielectric constants, respectively; *A* is the electrode area; *e* is the charge of the electron; *N*_D_ is the donor density; *V*_bias_ is the applied potential; *k*_B_ is the Boltzmann constant; and *T* is the temperature. Measurements of the electrochemical impedance between −0.45 and 2.25 V vs RHE at different frequencies from 100 to 1000 Hz were performed.

The Mott–Schottky plot for TiS_3_ in the KOH aqueous electrolyte has been reported for the first time (Figure 8d). A value of the flat band potential of −0.07 ± 0.04 V vs RHE was obtained. This value is similar to that obtained in Na_2_SO_3_ at pH = 9,^10^ which was reported to be −0.48 ± 0.02 V NHE. By using eq 2, this potential is 0.05 ± 0.02 V vs RHE, which is in reasonable agreement with the value obtained here in KOH taking into account the different pH of each electrolyte (9 vs 14).

Furthermore, the Mott–Schottky plots for some oxidized samples (ox-air-12 and ox-air-20) included in Figure 8d, yield similar *V*_fb_ values to that of the TiS_3_. This fact suggests that the influence of the amorphous TiO_2_ layer on the semiconductor capacity is not evident under the investigated conditions. Finally, capacitance results from TiO_2_ anatase nanoribbons have also been included in Figure 8d as a reference and its flat band potential obtained is 0.2 ± 0.01 V vs RHE (see also the zoom of that graph in Figure 8e). Therefore, it can be concluded that the position of the TiS_3_ flat band potential does not change if the TiO_2_ oxide shell is amorphous and thin enough, hinting that this structure does not significantly modify the capacitance of the semiconductor space charge layer. On the contrary, there were two samples (ox-air-90 and ox-Ar-240) whose Mott–Schottky plots do not converge, and the flat band potential could not be determined under the same experimental conditions either using the same equivalent circuit (Figure S7). This fact should be investigated in depth to clarify how the TiO_2_ thickness and structure affect the semiconductor/electrolyte interface capacitance, as previously proposed for perovskite solar cells by the identification of the optimal frequency ranges for the different layers.^66^

**Figure 9:**
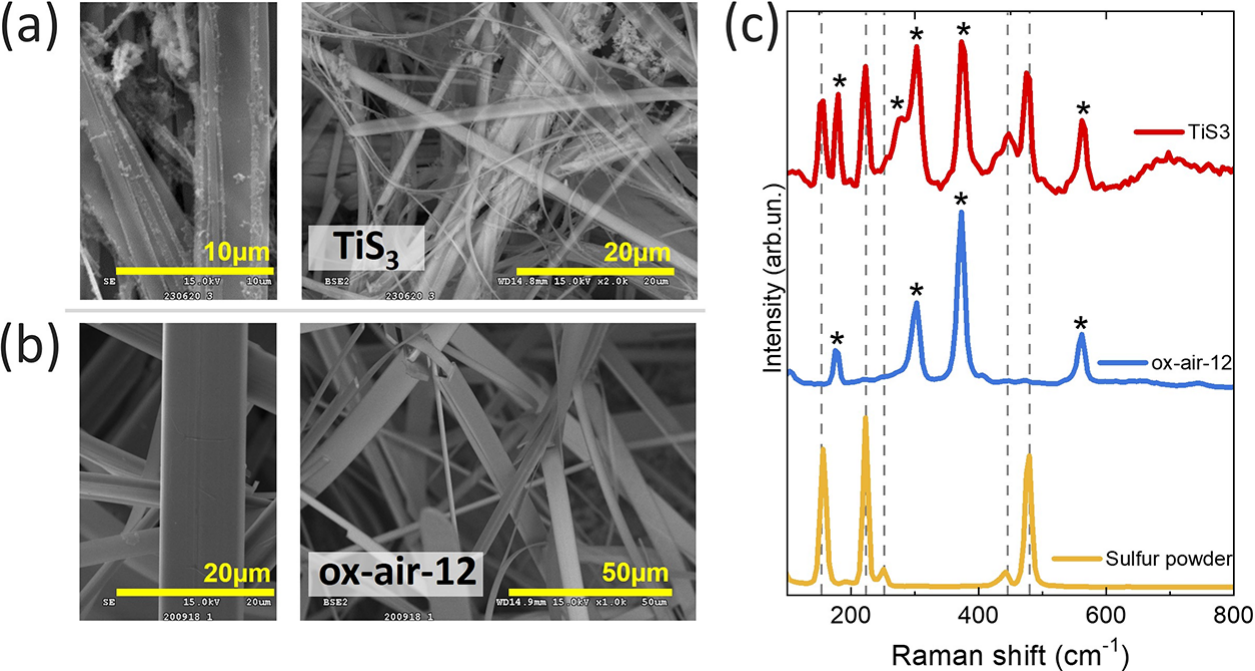
SEM images of (a) TiS_3_ and (b) ox-air-12 at different magnifications after the PEC experiments. (c) Raman spectra of TiS_3_ and ox-air-12 after the PEC measurements. For comparison, it is also shown the spectra of the sulfur powder collected from the ampules in which the TiS_3_ samples are synthesized. TiS_3_ Raman bands are indicated with a *.

After the PEC measurements, the unprotected TiS_3_ sample looks quite deteriorated, as shown in Figures S8 and S9, in which even some sulfur can be seen coming out of the TiS_3_ electrode during the PEC tests. Instead, ox-air-12 shows no difference between the before and after the PEC measurements, and no sulfur deposits were observed. Different characterization techniques were employed to prove the degradation degree of pristine TiS_3_ compared with the TiS_3_/TiO_2_ heterostructures. SEM images (Figure 9a,b) confirm the results observed at a glance (Figure S8). The pristine TiS_3_ nanoribbons appear degraded with some deposits stuck on top, whereas the morphology of the oxidized samples is well maintained. Raman measurements done on those same samples (Figures 9c and S9a,b) elucidate that those deposits are sulfur, expulsed of the samples during the PEC measurements in KOH. These results are in good agreement with the ones observed in the pictures shown in Figure S8, and with the XRD pattern reported in Figure S9c, which has been helpful in hypothesizing the sulfur exposure mechanism (eq S1). Those sulfur peaks were not observed for ox-air-12. After these findings, it was concluded that the oxide layer protects against degradation of the morphology and composition of the TiS_3_ nanoribbons.

The stability of ox-air-12 compared to pristine TiS_3_ has been further demonstrated by means of long-term chronoamperometry measurements and the stabilization of their photocurrents over time, as can be seen in the Supporting Information (see Figure S10 and the discussion there).

Given that the oxidation of sulfide minerals and compounds in alkaline media is well known,^67^ we believe that, in addition to protecting the surface of TiS_3_ with coatings like TiO_2_ as we proposed here, the presence of hole scavengers such as sulfites (SO_3_^2−^) and polysulfides (S_*x*_2−) in the electrolyte would contribute to the stabilization. Furthermore, reducing the KOH concentration would also enhance the stabilization of this sulfide compounds, including TiS_3_.

#### 3.4.1 Preliminary Results about the Use of Electrocatalysts to Improve the Charge Transfer Reactions at the TiS_3_/TiO_2_/KOH Interface

**Figure 10:**
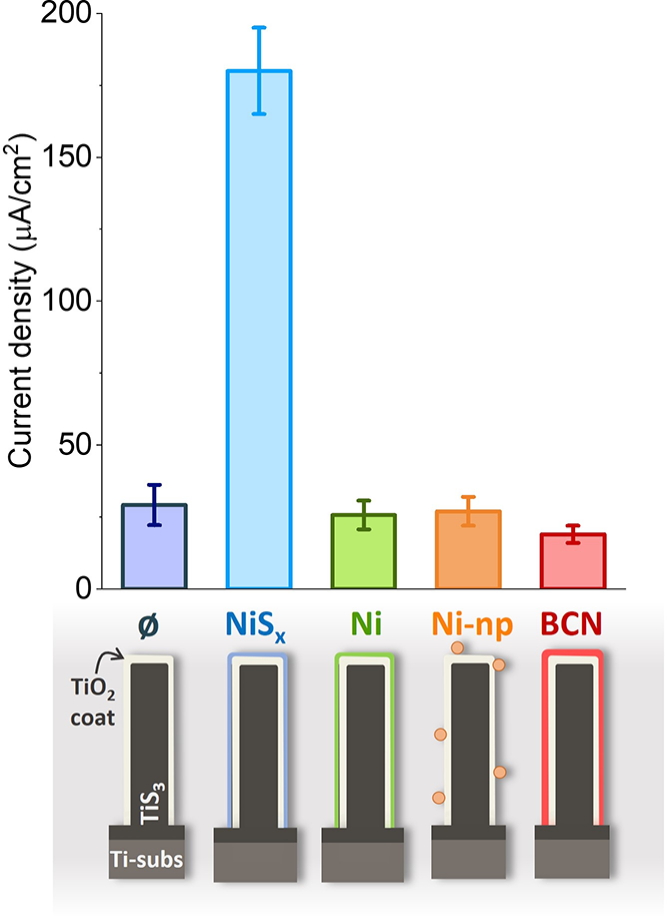
Top: Photocurrents obtained at 1.95 V vs RHE for samples oxidized in air for 12 min, without photoelectrocatalyst (ø), with NiS*_x_*, Ni thin film (4 nm thick), Ni nanoparticles (Ni-np), and BCN. Bottom: Schematic of the material.

Once the effectiveness of protecting TiS_3_ with TiO_2_ was demonstrated, some electrocatalysts were deposited on samples oxidized in air for 12 min, as these samples showed the best photocurrents (see Figure 8b). Figure 10 reports the photocurrents for all of the electrocatalysts tested under this research compared to a bare ox-air-12 sample.

First, based on the good results obtained for BCNs as electrocatalysts for the OER,^24,32^ it was decided to deposit them over the TiS_3_/TiO_2_ heterostructures. It was observed that the PE-CVD technique has an impact on the structure of the nanoribbons, in which a crystallization of amorphous TiO_2_ was noticed (see Figure S11). It has been already reported that the PE-CVD technique used to grow BCN layers, can alter the structure of the sample.^24,33^ Particularly, in one of our previous works, the transformation of anatase TiO_2_ nanoribbons to rutile TiO_2_ due to the high temperatures reached inside the ampules was discussed.^24^ Therefore, due to the crystallization of the TiO_2_ structure, the positive effect of BCN as an electrocatalyst is blurred and there is a decrease of the ox-air-12 photocurrent. This can be explained as the electron–hole pairs recombine before reaching the electrode–electrolyte interface, as discussed before (Figure 8c).

After that, Ni-based electrocatalysts were used. Ni thin films of 4 nm were deposited by thermal evaporation, and Ni nanoparticles (500 nm diameter) were deposited by bathing the samples in a solution of Ni nanoparticles in heptane. The values of the photocurrents are shown in Figure 10, in which it can be appreciated that in both cases, they are similar to the one obtained for the ox-air-12 electrode. The absence of a positive effect has been assigned to bad adherence of both the Ni films and Ni nanoparticles to the TiO_2_ surface. SEM and EDX mappings were performed for the sample with the Ni film before and after the PEC tests (Figure S12). It is noticed that the amount of Ni in the sample decreases by about 50%, which is evidence that the Ni layer has detached from the ox-air-12 sample.

Finally, it was attempted to improve the adherence of the Ni films by sulfurizing at 200 °C for 4 h. NiS_*x*_ is a material that has been previously reported to be a good electrocatalyst for the OER. This electrocatalyst transforms from NiS_*x*_ to NiOOH or Ni(OH)_2_ phases, which are well known for being active phases for the OER.^68−70^ That positive effect was evidenced by the considerable increase in the photocurrent of the ox-air-12 electrode. After the experiments, XPS and Raman characterization of this sample were performed, observing the presence of these phases, as well as peaks ascribed to TiS_3_ and TiO_2_, confirming the good stability of these electrodes (see Figures S13 and S14). The advantage of this electrocatalyst over the others is that it does not alter the amorphous oxide layer obtained by thermal oxidation in air, as is the case of BCN layers. Moreover, it does not detach from the surface after photoelectrochemical measurements.

The results described in this section indicate that with the correct electrocatalyst, the photoelectrochemical properties of the TiS_3_/TiO_2_ electrodes could be increased.

## 4 Conclusions

In this study, we examined a promising heterostructure for photoelectrochemical (PEC) water splitting, which consists of TiO_2_-coated TiS_3_ nanoribbons. We discovered a quick and effective method to protect TiS_3_ by using a TiO_2_ shell. This involved the annealing of TiS_3_ at 300 °C in different oxidizing atmospheres, such as air and Ar with some traces of residual oxygen. The resulting TiO_2_ shells were either amorphous or crystalline, depending on the type of oxidizing atmosphere used (air or Ar with traces of residual oxygen, respectively). Our PEC measurements validate the effectiveness of the TiO_2_ coating in protecting TiS_3_ from corrosion of strongly alkaline electrolytes (such as KOH) while still allowing the transfer of photogenerated charge carriers to the electrode/electrolyte interface. We found that an amorphous TiO_2_ layer was better suited for this purpose, as crystalline TiO_2_ had a tendency to worsen the photoelectrochemical current. Overall, we found that a thin and amorphous TiO_2_ shell improved the photocurrents and stabilization potential of TiS_3_ without altering the flat band potential. Additionally, we tested different electrocatalysts on the TiS_3_/TiO_2_ heterostructure and found that NiS_*x*_ thin films produced the best results.

## Supporting Information Available

Detailed description of the thermal annealing treatment and table with the synthesis condition of the samples; schematic of the photoelectrochemical cell; XPS fittings for samples oxidized under different conditions and table with the fitting parameters; XPS spectra in the 165–175 eV region; calculated percentages of the TiS_3_ and TiO_2_ contents based on the density of Ti; Raman spectra of TiS_3_ through the width of a nanoribbon; Tauc plottings for the diffuse reflectance measurements and energy band gap values; Mott–Schottly plots for two samples; photographs that evidence the degradation of TiS_3_ in KOH, and stability measurements of the oxidized samples compared to pristine TiS_3_; evidences of the synthesis of BCN; and evidences of the deposition of NiS_*x*_ and Ni and their stability (PDF)
