# Supplementary material for: Protecting TiS3 Photoanodes for Water Splitting in Alkaline Media by TiO2 Coatings
Source: ACS Appl Mater Interfaces. 2024 Jun 19;16(26):33696–709. doi: 10.1021/acsami.4c07404 (PMC11231970; doi:10.1021/acsami.4c07404)
Supplement: Supplementary file 1 — am4c07404_si_001.pdf [file am4c07404_si_001.pdf]

Supporting Information

Protecting TiS_3 photoanodes for water splitting in alkaline media by TiO_2 coatings

Nuria Jiménez-Arévalo [a,], Eduardo Flores [b], Alessio Giampietri [c,&], Marco Sbroscia [c], Maria Grazia Betti [c], Carlo Mariani [c], F. Javier García-García [d], José R. Ares [a], Fabrice Leardini [a,e], Isabel J. Ferrer [a,e]*

[a] Departamento de Física de Materiales, Universidad Autónoma de Madrid, Campus de Cantoblanco, E-28049 Madrid, Spain.
[b] Departamento de Física Aplicada, Centro de Investigación y Estudios Avanzados, 97310, Mérida, México.
[c] Dipartimento di Fisica, Università di Roma 'La Sapienza', I-00185, Italy.
[d] ICTS-Centro Nacional de Microscopía Electrónica, Universidad Complutense de Madrid, E-28040 Madrid, Spain
[e] Instituto Nicolás Cabrera (INC), Universidad Autónoma de Madrid, Campus de Cantoblanco, E-28049 Madrid, Spain.
* Corresponding author: nuria.jimenezarevalo@uniroma1.it

Synthesis and preparation

TiS_3 oxidation

TiS_3/TiO_2 heterostructures are obtained by a thermal annealing treatment of the TiS_3 samples. The thermal annealing process is carried out using a tubular quartz furnace placed onto two rails that allows its horizontal movement. A diagram of the furnace movement can be seen in Figure S1a. The temperature of the sample is controlled by a thermocouple placed close to the sample to monitor its temperature in each moment.

The oxidation procedure takes place in three steps (see Figure S1b for the heating profile and the schematic of the different steps):

1- A first temperature ramp is programmed to reach the oxidation temperature (300 °C) with the furnace far away from the sample. At this point the sample is still at room temperature.
2- Once the furnace reaches 300 °C, it is moved a distance that permits to place the sample on its centre. At this point, the temperature of the sample starts to increase until it reaches 300°C.
3- Once the desired oxidation time has passed (oxidation time is a variable that affects the thickness of the oxide layer), the furnace is moved again away from the sample. This causes the sample to cool down to room temperature.

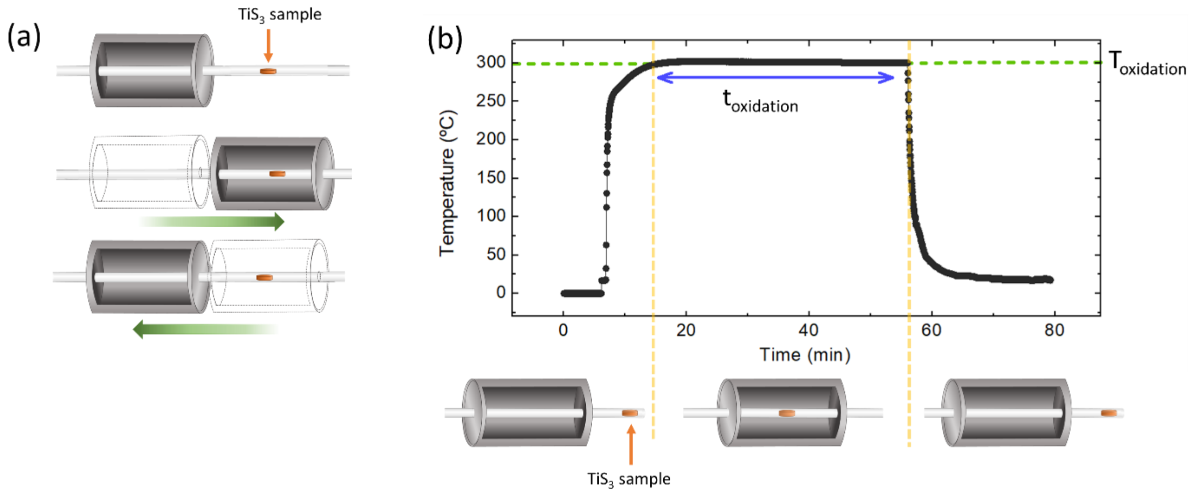

Figure S1. (a) Schematic of the different movements that the furnace can do with respect to the sample. (b) Temperature profile of the sample measured with a thermocouple during the oxidation process and the scheme of the relative position of the sample to the furnace. $T_{oxidation}$ and $t_{oxidation}$ refer to the oxidation temperature and time, respectively.

As mentioned in the main text, samples have been named according to their oxidation time and the oxidation atmosphere. Table S1, summarises the name of the samples and their oxidation atmosphere and time.

Table S1. Name of the samples under investigation in this work and the oxidation atmosphere and time employed for their treatment. The oxidation temperature was set at 300°C for all samples.

Name	Oxidation atmosphere	Oxidation time (min)
ox-Ar-20	An Argon flow of 100 sccm with residual traces of air, after 1h of purging with an Ar flow of 200sccm.	20
ox-Ar-54		54
ox-Ar-140		140
ox-Ar-230		230
ox-Ar-240		240
ox-air-12	air	12
ox-air-20		20
ox-air-30		30
ox-air-90		90

Characterization

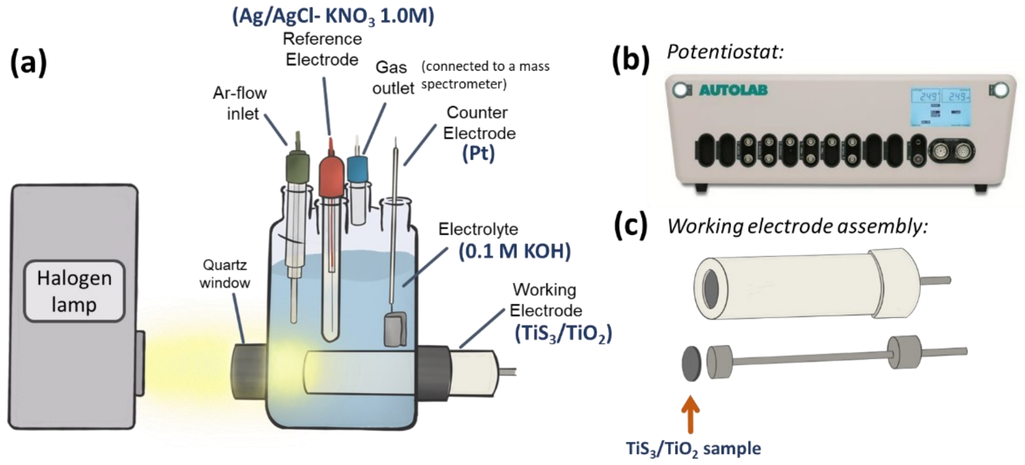

Figure S2. (a) Schematic diagram of the photoelectrochemical cell used in this work. (b) Picture of the potentiostat to which the three electrodes are connected. (c) Working electrode holder assembly.

Results and discussion

XPS results

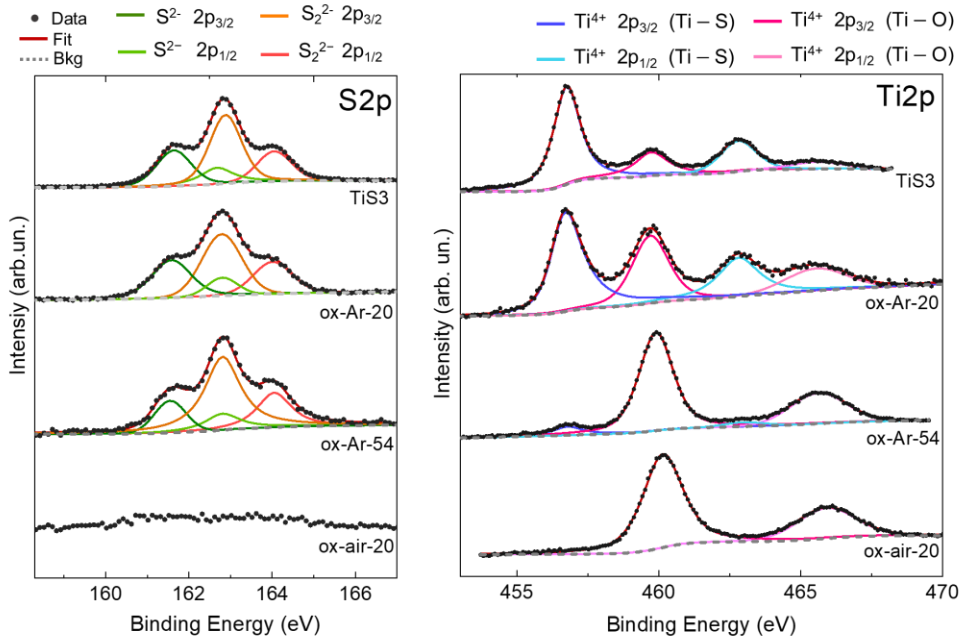

Figure S3. S2p (left panel) and Ti2p (right panel) XPS spectra for TiS_3, ox-Ar-20, ox-Ar-54 and ox-air-20; the data are stacked along the vertical axis for clarity. Experimental data (dots), Shirley background is represented by a grey dotted line, the total fitting curve for each measurement is in red and the single fitting components (coloured lines) as described in the legend.

Table S2. Position (binding energy, BE), full peak width at a half maximum (FWHM), area of the peak and relative intensities of the core levels for TiS_3, ox-Ar-20, ox-Ar-54 and ox-air-20 samples.

Sample	Core level	Core level component		BE (eV)	FWHM (eV)	Area	Single Peak Relative intensity (%)	Components Relative Intensity (%)
**TiS_3**	S2p	S^{2-}	$2p_{3/2}$	161.4	0.99	233.3	24.0	37.4
			$2p_{1/2}$	162.7	0.93	130.0	13.4	
		S_2^{2-}	$2p_{3/2}$	162.9	0.91	424.5	43.7	62.6
			$2p_{1/2}$	164.1	0.97	182.9	18.9	
	Ti2p	Ti^{4+} (Ti-S)	$2p_{3/2}$	456.8	1.12	588.4	56.3	76.1
			$2p_{1/2}$	462.8	1.48	207.2	19.8	
		Ti^{4+} (Ti-O)	$2p_{3/2}$	459.7	1.39	187.5	17.9	23.9
			$2p_{1/2}$	465.6	2.22	62.7	6.0	
**ox-Ar-20**	S2p	S^{2-}	$2p_{3/2}$	161.6	1.12	122.5	28.0	37.2
			$2p_{1/2}$	162.8	0.86	40.0	9.2	
		S_2^{2-}	$2p_{3/2}$	162.8	1.12	180.0	41.2	62.8
			$2p_{1/2}$	164.0	1.08	94.7	21.7	
	Ti2p	Ti^{4+} (Ti-S)	$2p_{3/2}$	456.8	1.36	328.0	39.7	60.0
			$2p_{1/2}$	462.8	1.71	167.0	20.2	
		Ti^{4+} (Ti-O)	$2p_{3/2}$	459.7	1.52	225.9	27.4	40.1
			$2p_{1/2}$	465.5	2.53	104.8	12.7	
**ox-Ar-54**	S2p	S^{2-}	$2p_{3/2}$	161.6	0.89	12.5	14.3	25.7
			$2p_{1/2}$	162.8	1.00	10.0	11.4	
		S_2^{2-}	$2p_{3/2}$	162.8	1.03	46.0	52.3	74.4
			$2p_{1/2}$	164.1	0.97	19.4	22.1	
	Ti2p	Ti^{4+} (Ti-S)	$2p_{3/2}$	456.8	1.24	45.1	5.7	8.4
			$2p_{1/2}$	463.0	1.69	21.3	2.7	
		Ti^{4+} (Ti-O)	$2p_{3/2}$	459.9	1.24	510.6	64.7	91.6
			$2p_{1/2}$	465.6	2.31	212.0	26.9	
**ox-air-20**	Ti2p	Ti^{4+} (Ti-O)	$2p_{3/2}$	460.1	1.67	517.0	71.4	100
			$2p_{1/2}$	465.9	2.41	207.4	28.6	

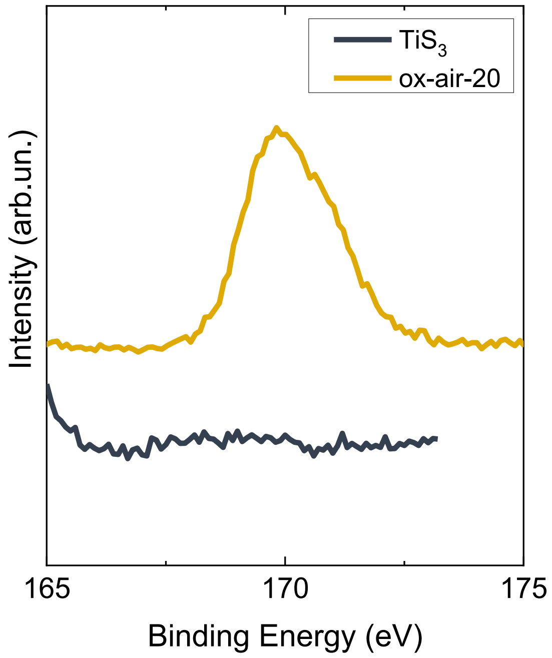

Figure S4. XPS spectra in the region from 165 eV to 175 eV for TiS_3 and ox-air-20 samples.

EDX Mapping

Table S3 gives information about the mass percentage of Ti and the density of TiS_3 and TiO_2. By multiplying the mass percentage and the density we obtain the density of Ti in both compounds. As it can be seen, the density of Ti in TiO_2 is 2.5 folds higher than the density of Ti in TiS_3. This result explains well why the EDX Ti signal is lower in the region ascribed to the TiS_3 than in the TiO_2 ones.

Table S3. Mass percentage of Ti, density and density of Ti in TiS_3 and TiO_2

Compound	Mass percentage of Ti (%)	Density (g/cm^3)	Ti density (g/cm^3)
TiS_3	33	3.21	1.06
TiO_2	60	4.34	2.60

Raman spectra of TiS_3 through the width of a nanoribbon.

Figure S5b shows the Raman spectra in the edge and centre of one nanoribbon of TiS_3. The TiS_3 peaks positioned in 175 cm^{-1}, 300 cm^{-1}, 370 cm^{-1} and 563 cm^{-1} [1,2], named as peak 0, 1, 2 and 3, respectively can be observed in both spectra. In the spectrum recorded at the edge of the nanoribbon, a peak centred in 270 cm^{-1} arises (named as peak *). This peak has also been reported to be from TiS_3 [3].

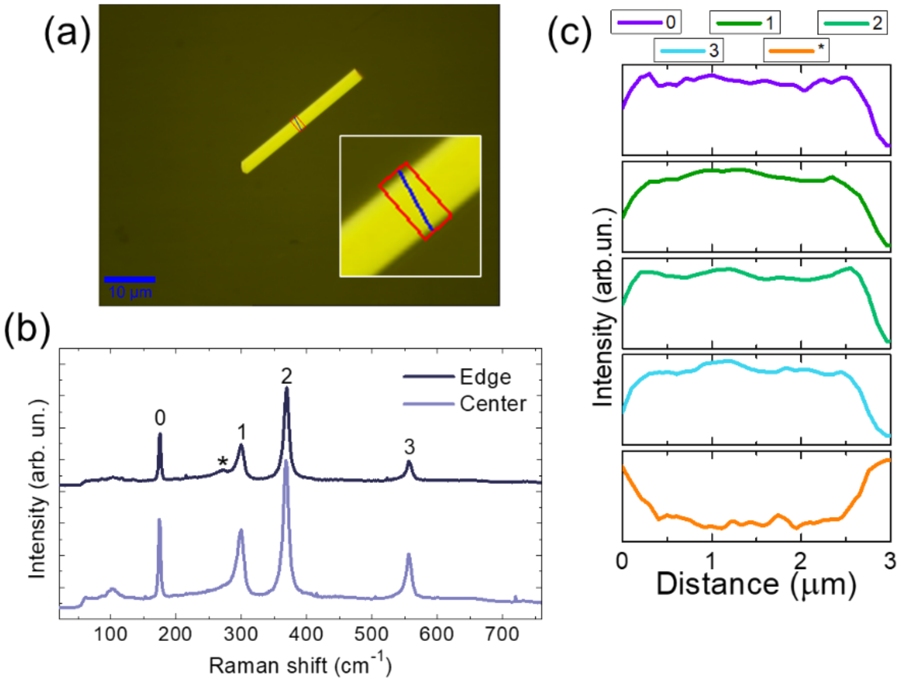

Figure S5. (a) Optical microscopy image for a TiS_3 nanoribbon. (b) Raman spectra on the edge and centre of the nanoribbon. (c) Evolution of the intensity of the peaks marked in (b) through the width of the nanoribbon (blue line in (a)).

Optical characterization of the electrodes. Kubelka-Munk function.

Diffuse Reflectance spectroscopy (DRS) is a technique widely used to optically characterize powder materials, natural compounds and minerals including information about their band gap energy. From the DRS data the optical absorbance spectrum can be obtained using some complex formalism. However, usually a good approximation to be used is the Kubelka-Munk function which is proportional to the optical absorbance and is defined by the following equation [4]:

$$F(R) = \frac{(1-R)^2}{2R}$$

This function *F(R)* is frequently used to determine the value of Eg by applying the Tauc approximation for direct and indirect allowed electronic transitions responsible for the optical absorption threshold [5] by plotting $[F(R) \cdot h\nu]^n \; vs \; (h\nu)$ being *n=0.5* for indirect and *n=2* for direct transitions.

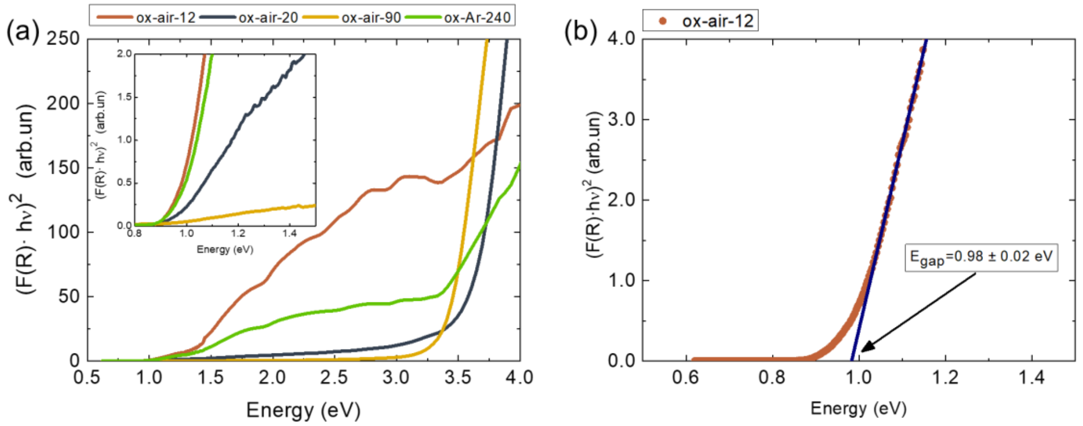

Figure S6. (a) Tauc plot for samples ox-air-12, ox-air-20, ox-air-90 and ox-Ar-240. Inset is a zoom between 0.8 eV and 1.5 eV. (b) Fitting made to ox-air-12 data and the corresponding band gap energy value.

Table S4. Values and uncertainties of the optical band gap energies (corresponding to the TiS_3 phase) of the samples shown in Figure6. The average value is also shown.

Sample	E_g (eV)	ΔE_g (eV)
ox-air-12	0.98	0.02
ox-air-20	0.95	0.01
ox-air-90	0.92	0.01
ox-Ar-240	0.97	0.03
Average:	0.96	0.03

Photoelectrochemical results

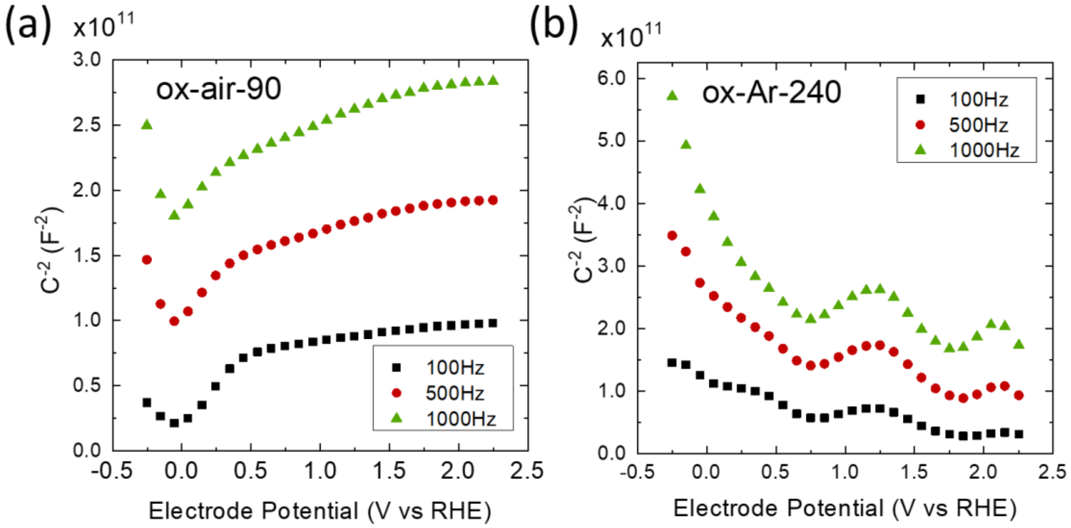

Figure S7. Mott-Schottky plot of (a) b ox-air-30 and (b) ox-Ar-240 samples at three representative frequencies.

TiS_3 degradation

TiS_3 samples were immersed in KOH media to prove their degradation. SEM and Raman characterizations comparing pristine TiS_3 with ox-air-12 are given in Figure 9 in the main text. Here, additional characterizations are reported.

Figure S8 shows photographs of the pristine TiS_3 sample and ox-air-12 before, during and after the photoelectrochemical measurements using 0.1M KOH. The degradation of the TiS_3 sample compared to the oxidized sample was also observable in the bare eye (see pictures in Figure S8), in which the existence of sulfur deposits (in yellow and indicated with an arrow) is clear. The final state of the electrode is different for the bare TiS_3 and the protected one. The TiS_3 electrode looks thoroughly degraded; meanwhile, the ox-air-12 (the sample that has been oxidized the lowest amount of time) appears unaltered (as also proved by SEM in Figure 9b in the main text).

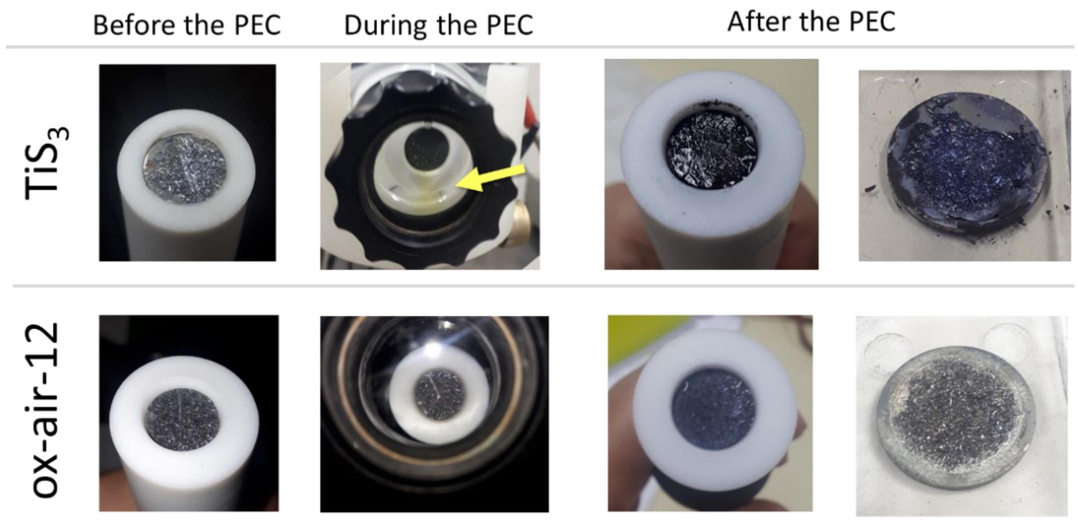

Figure S8. Photographs of the TiS_3 and ox-air-12 samples before, during and after the PEC in 0.1M KOH aqueous electrolyte. Notice the yellow sulfur drops falling from the TiS_3 during the PEC measurements (indicated with an arrow).

Figure S9a shows an optical microscopy image of the TiS_3 sample after the photoelectrochemical characterization. It can be observed that the nanoribbons are agglomerated and bounded by some deposits. The formation of sulfur deposits was confirmed by measuring the Raman spectra in several points, as indicated in Figure S9a, as can be observed in Figure S9b. Some areas show the presence of pure TiS_3 (such as point 1), but other (point 4) evidence the presence of sulfur peaks similar to the ones obtained by analysing the sulfur powder used to sulfurize the Ti disks (see also Figure S9b).

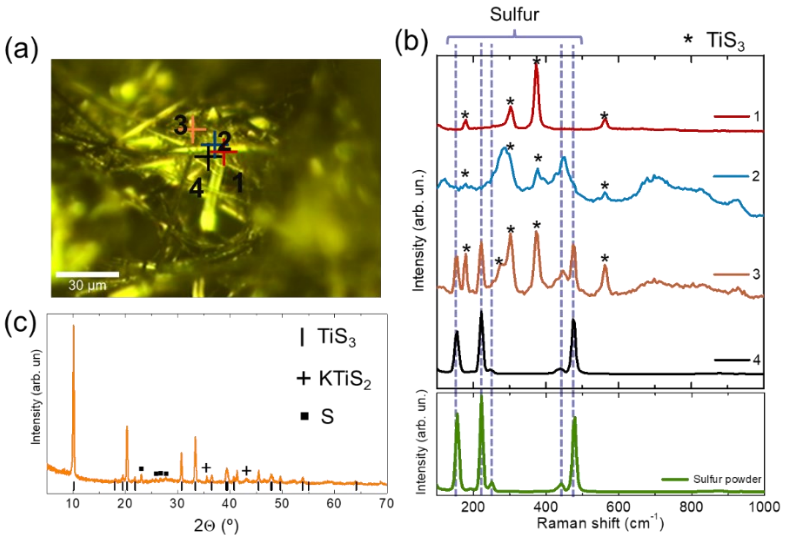

Figure S9. (a) Optical microscopy image of a TiS_3 electrode after the PEC measurement and (b) Raman spectra in the indicated points. TiS_3 peaks are indicated with * and sulfur peaks with a grey dotted line. Sulfur spectrum was recorded on the sulfur powder used for the sulfurization of the Ti substrates. (c) XRD diffractogram of that sample after the PEC. TiS_3 peaks correspond to PDF 00-015-0783, $KTiS_2$ peaks to PDF 00-023-1364 and S peaks to PDF 00-024-0733.

Additional XRD characterization was conducted. TiS_3 peaks were observed, as well as peaks due to $KTiS_2$ and S, as indicated in Figure S9c. These results were used to propose a possible degradation mechanism of the TiS_3 in the presence of KOH, which would be:

$$TiS_3 + KOH \rightarrow KTiS_2 + S + OH^- \quad \text{(equation S1)}$$

Chronoamperometry measurements

Additional measurements have been carried out with different couples of samples (air-oxidized TiS_3 vs pristine TiS_3) specifically synthesised to compare the stability of TiS_3/TiO_2 structures to that of pristine TiS_3. The main conclusions obtained can be resumed in Figures S10 a and b.

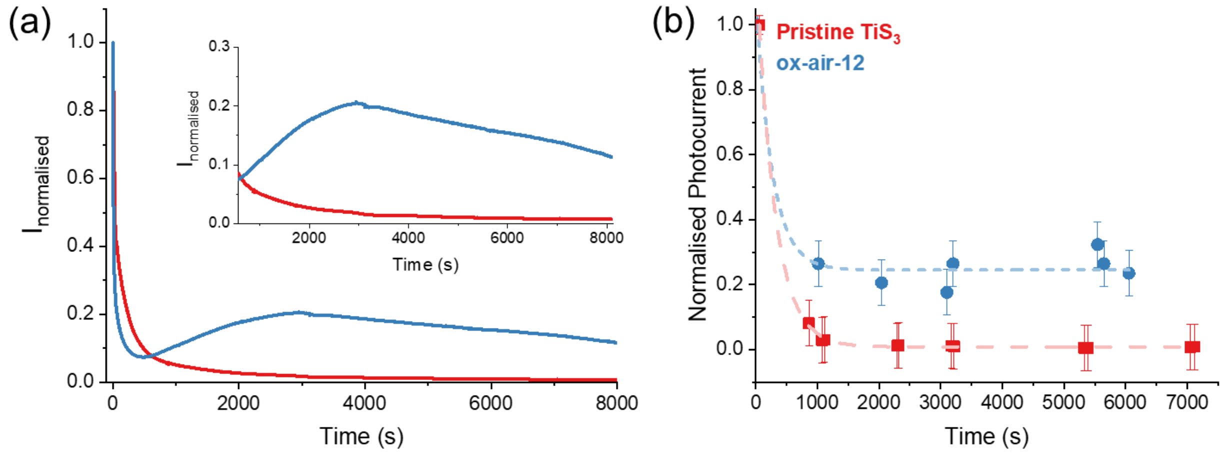

Figure S10. (a) Chronoamperometry normalized current density of pristine and oxidised (ox-air-12) TiS_3 samples in aqueous KOH 0.1 M, at 1,95V vs RHE under the illumination of a halogen lamp (65mW/cm^2). (b) Normalized photocurrent density of pristine and oxidised (ox-air-12) TiS_3 samples in aqueous KOH 0.1 M, at 1,95V vs RHE under the illumination of a halogen lamp (65mW/cm^2).

Figure S10a shows the time evolution of the normalized current to the first value at time t=0 sec during 2 hours of illumination for both samples, pristine and oxidized (similar conditions of ox-air-12) TiS_3. Firstly, a sharp current fall is observed in the first seconds of the chronoamperometry, which is more pronounced in the oxidized sample. However, after 300 seconds, the normalized current of the oxidized sample changes its trend and starts to increase, crossing the plot of the one of pristine TiS_3. As can be observed in the inset of Figure S10a, normalized current decreases with time down to reach 1% of the initial value, while that of the oxidized sample keeps above 12% of its initial value for more than 2 hours.

Concerning the photocurrent, the effect of the oxidation layer is similar to that of the total current (see Figure S10b). The pristine and oxidized sample photocurrents, measured at different times during the chronoamperometry and normalized to those at t=0, are shown in Figure S10b. As can

be seen, the normalized photocurrent of pristine TiS_3 decreases to 0.8% of its initial value in a few minutes, while that of the oxidized sample keeps at 24% of its initial one.

Overall, both curves demonstrate that the oxidation treatment improves the photoelectrochemical behavior and has an indubitable protecting effect against the deterioration of TiS_3.

Preliminary results about the use of electrocatalysts to improve the charge transfer reactions at the $TiS_3/TiO_2/KOH$ interface.

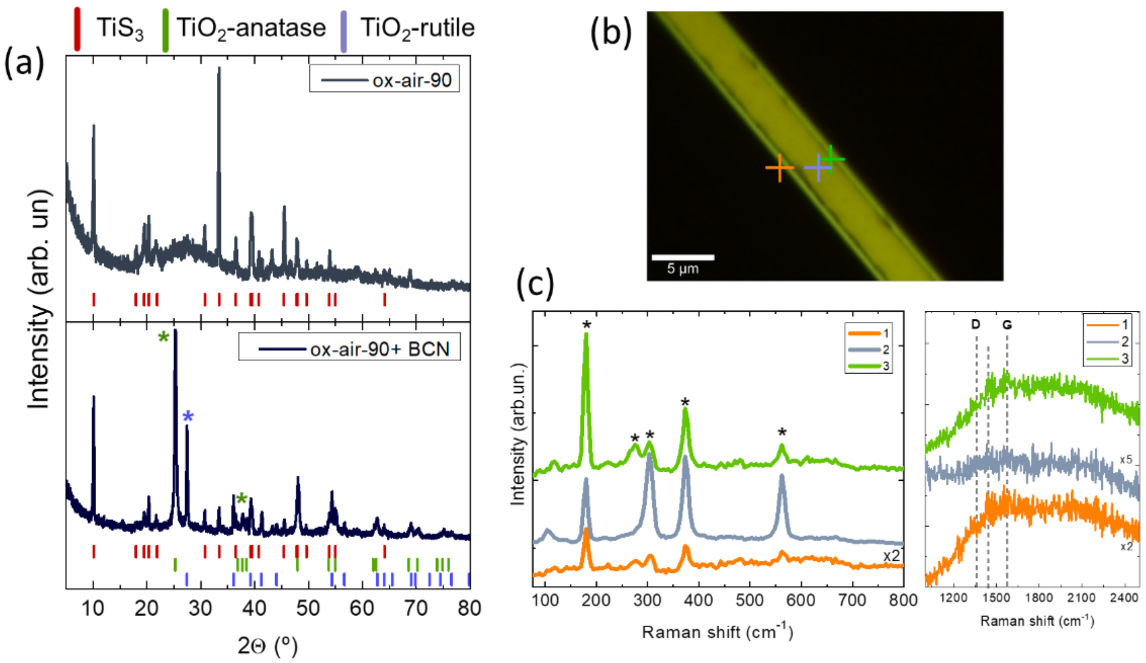

Figure S11. (a) XRD diffractogram for TiS_3 samples oxidised in air for 90 minutes one without BCN and the other one after the BCN deposition. TiS_3, TiO_2-anatase, and TiO_2-rutile peaks correspond to PDF 00-015-0783, PDF 01-071-1167, and PDF 01-073-2224 files. Asterisks indicate the most intense peak of each phase that appear after the BCN growth. Colours of the asterisks correspond to the legend. (b) Optical microscopy image of sample ox-air-30 with BCN deposited. (c) Raman spectra (on the marked with cross points in (b)) of the air oxidated sample (4.5x zoom in the 1000-2200cm-1 region)

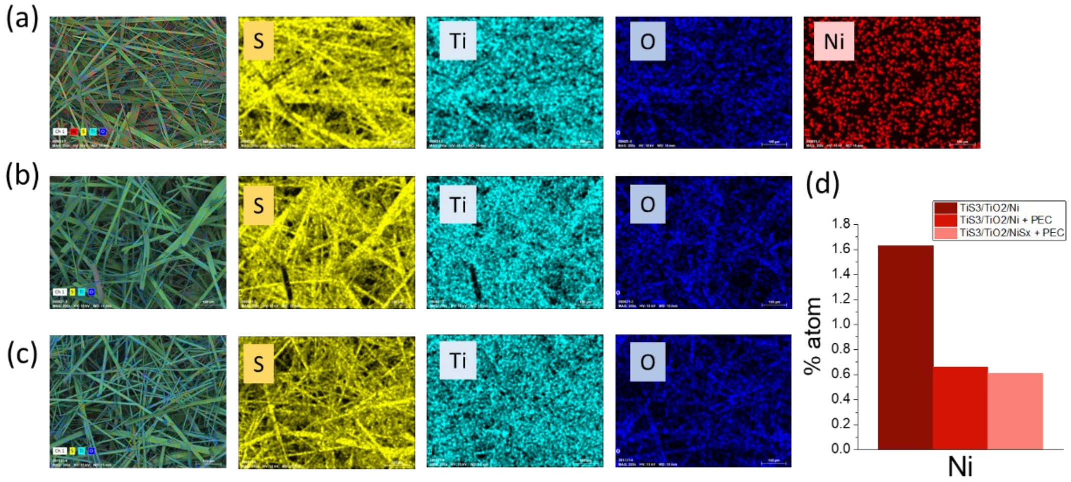

Figure S12. EDX mappings of (a) Ni deposited on TiS_3/TiO_2 heterostructures before the PEC, (b) after the PEC, and (c) NiS_x deposited on TiS_3/TiO_2 heterostructures after the PEC. (d) Ni content in these three samples.

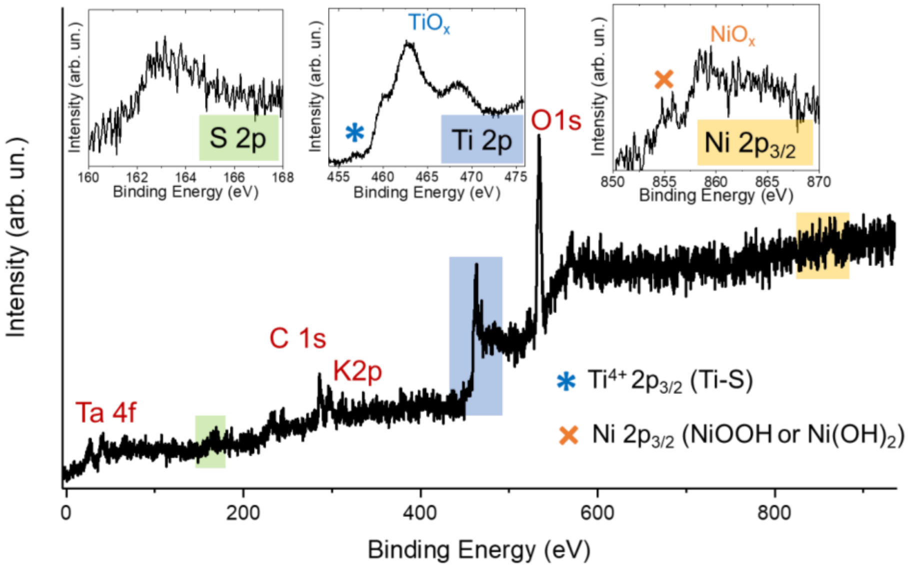

Figure S13. XPS spectra of the sample ox-air-12 after the photoelectrochemical characterization. First it can be observed the presence of K 2p and an intense O 1s peak due to the exposure of the samples to KOH. At the same time C 1s is observed probably due to the presence of atmosphere contamination that was not removed after an annealing over night at 120ºC. Ta 4f peaks are due to the Ta clips used to hold the sample onto the Mo sample holder. The peaks of interests (S 2p, Ti 2p and Ni 2p) are inserted as insets on the top part of the figure. It can be noticed the presence of an S 2p component. Nevertheless, this component is not due to sulfur contamination, but the presence of TiS_3 on the topmost 7nm of the sample, which indicates that the oxide layer is thin. It could also be observed some peaks ascribed to the presence of TiO_x compounds, as evidenced on the figure, and Ni due to the formation of NiOOH or $Ni(OH)_2$ compounds, as well as NiO_x [6].

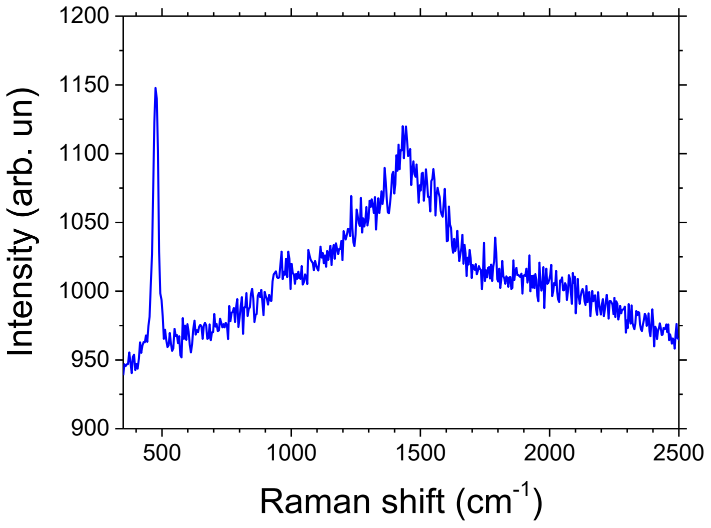

Figure S14. Raman spectrum of the sample ox-air-12 after the photoelectrochemical characterization. It can be observed a sharp peak centered at 480 cm^{-1}, which is indicative of NiOOH formation [6], as well as a broad peak at 1450 cm^{-1}, which is ascribed to the second-order two-magnon band of NiO [7].

References

[1] N. Jiménez-Arévalo *et al.*, "Ultrathin Transparent B-C-N Layers Grown on Titanium Substrates with Excellent Electrocatalytic Activity for the Oxygen Evolution Reaction," *ACS Appl Energy Mater*, vol. 3, no. 2, pp. 1922–1932, 2020, doi: 10.1021/acsaem.9b02339.

[2] A. S. Pawbake *et al.*, "Temperature-Dependent Raman Spectroscopy of Titanium Trisulfide (TiS_3) Nanoribbons and Nanosheets," *ACS Appl Mater Interfaces*, vol. 7, no. 43, pp. 24185–24190, 2015, doi: 10.1021/acsami.5b07492.

[3] P. Gard, F. Cruege, C. Sourisseau, and O. Gorochov, "Single-Crystal Micro-Raman Studies of ZrS_3, TiS_3 and several $Zr_{1-x}Ti_xS_3$ compounds ($0<x<=0.33$)," *Journal of Raman Spectroscopy*, vol. 17, no. 3, pp. 283–288, 1986.

[4] A. B. Murphy, "Modified Kubelka-Munk model for calculation of the reflectance of coatings with optically-rough surfaces," *J Phys D Appl Phys*, vol. 39, pp. 3571–3581, 2006, doi: 10.1088/0022-3727/39/16/008.

[5] J. Tauc, R. Grigorovici, and A. Vancu, "Optical Properties and Electronic Structure of Amorphous Germanium," *Physica Status Solidi (b)*, vol. 15, pp. 627–637, 1966, doi: 10.1002/PSSB.19660150224.

[6] H. Radinger, P. Connor, S. Tengeler, R. W. Stark, W. Jaegermann, and B. Kaiser, "Importance of Nickel Oxide Lattice Defects for Efficient Oxygen Evolution Reaction", *Chem. Mater.* vol. 33, pp. 8259–8266, 2021, doi: 0.1021/acs.chemmater.1c02406

[7] E. Aytan, B. Debnath, F. Kargar, Y. Barlas, M. M. Lacerda, J. X. Li, R. K. Lake, J. Shi, and A. A. Balandin, "Spin-phonon coupling in antiferromagnetic nickel oxide", *Applied Physics Letters* , vol. 111, pp. 252402, 2017, doi: 10.1063/1.5009598
